# Exploration of the Prognostic Role of Apoptosis‐Related Genes in Glioblastoma

**DOI:** 10.1155/sci/8727203

**Published:** 2025-12-16

**Authors:** Hailong Wang, Lijun Yang, Yansong Lu

**Affiliations:** ^1^ Department of Neurosurgery, Jiangshan People’s Hospital, Quzhou, Zhejiang, China; ^2^ Department of Neurosurgery, People’s Hospital of Xinchang, Shaoxing, Zhejiang, China

**Keywords:** apoptosis-related genes, glioblastoma, intercellular communication, prognostic model, tumor immunity

## Abstract

**Background:**

Glioblastoma (GBM) is the most common and aggressive malignant neoplasm in the central nervous system. Apoptosis is crucial in the genesis, progression, and management of tumors. Nevertheless, the influence of apoptosis‐associated genes on GBM prognosis is unclear.

**Methods:**

Transcriptome data and single‐cell sequencing data were obtained from TCGA, CGGA, and GEO databases. Differential genes related to apoptosis were screened using the limma software, and an apoptosis‐related gene prognostic model (apoptosis signature [AS] model) was constructed through univariate Cox analysis under the optimization of 101 machine learning algorithm combinations. Validation analyses were conducted using bioinformatics tools.

**Results:**

A notable divergence in the expression levels of genes associated with programed cell death was identified when comparing GBM neoplastic tissues to their surrounding non‐neoplastic counterparts. They were closely related to the prognosis of GBM patients. BRCA1, CHEK2, and IKBKE genes exhibited elevated levels of expression within neoplastic tissues and were identified as risk factors for prognosis, while ZMYND11, MAPK8, and RPS3 genes were highly expressed in adjacent nontumor tissues as protective factors. The AS model demonstrated good predictive performance across multiple datasets, showing a higher concordance index (*C*‐index) value compared to conventional indicators of outcome. Moreover, the correlation coefficient between HSPB1 and the risk score associated with the AS model was positive, with a value of 0.75 (*p* < 2.2e–16).

**Conclusions:**

An apoptosis‐related gene prognostic model (AS model) with high predictive performance was constructed and had close associations with the tumor immune microenvironment and intercellular communication. The HSPB1 had a good predictive effect on GBM prognosis.

## 1. Background

Glioblastoma (GBM) is a type of neoplasm within the central nervous system and the predominant primary cerebral malignancy, presenting a considerable obstacle in the field of neuro‐oncology. Despite the current standard treatment described by Roger Stupp et al. [1] 16 years ago, the prognosis remains poor. Despite continuous efforts in basic, translational, and clinical research, long‐term survival rates have only changed marginally [2]. The outlook for patients is exceedingly grim, with a median duration of life generally not exceeding 15 months [3] and a 1‐year survival probability of merely 41.4%. For those experiencing recurrence, the 1‐year survival probability remains at 41.4%, while the 5‐year survival probability plummets to 6.8% [4]. Established unfavorable indicators of prognosis encompass advanced age, suboptimal functional status, and incomplete excision. The survival rates were more favorable for younger patients (≤60 years), especially women, while older patients faced poorer outcomes [5]. Supramaximal resection could significantly prolong the median overall survival (OS) and progression‐free survival (PFS), especially for newly diagnosed GBM [6]. Additionally, molecular characteristics such as mutations in the IDH1 and IDH2 genes and methylation of the MGMT gene are linked to a more auspicious prognosis [2, 7].

Apoptosis, which is a form of programed cell demise, is pivotal in the genesis of tumors, their advancement, and therapeutic interventions. Aberrant expression of apoptosis‐related genes is intimately linked to the oncogenesis of diverse neoplasms and could influence the susceptibility of neoplastic cells to therapeutic regimens [8]. Certain research has indicated that the manifestation and governance of genes implicated in apoptosis may be inextricably connected to the virulence, therapeutic refractoriness, and prognostic outcome of patients with GBM. For instance, the methylation profile of the MGMT gene has been observed to correlate with the longevity of GBM patients, where individuals exhibiting favorable methylation patterns tend to experience extended survival durations [9]. The expression patterns of apoptosis‐related genes in GBM involve multiple signaling pathways and molecular mechanisms. Recent investigations have revealed an upregulation of CARD16 in malignant gliomas, particularly in GBM, where it impedes apoptosis through the ignition of the NF‐κB signaling cascade, thereby fostering the proliferation of tumor cells. Furthermore, CARD16 regulates apoptosis by affecting the FOXO1/TRAIL axis, and the amplification of FOXO1 expression can markedly diminish the oncogenic potential of GBM and enhance survival probabilities [10]. Infiltrating plasma cells secrete IgG that binds to FcγRIIA on GBM stem cells (GSCs), stimulating the PI3K‐AKT‐mTOR signal transduction cascade within GSCs, maintaining their self‐renewal, and leading to malignant progression of the tumor [11, 12].

Despite recent progress in therapeutic strategies, the high recurrence rate and treatment resistance of GBM remain major clinical challenges [13]. Research indicates that in GBM cells, apoptosis triggered by Selinexor is constrained by autophagy, and the suppression of autophagy can augment the susceptibility of GBM cells to Selinexor‐induced cell demise. Combination therapy with Selinexor and the autophagy inhibitor chloroquine increases GBM cell death and prolongs patient survival [14]. Additionally, a molecule named Gliocidin disrupts nucleotide balance by reducing intracellular guanine nucleotide levels, inhibiting IMPDH2 enzyme activity, leading to decreased DNA synthesis ability and subsequent cell death. It has potent blood–brain barrier permeability and can slow tumor progression and significantly improve survival rates in mouse models [15]. The small molecule compound THTMP regulates the activity of Bcl‐2 family proteins through the p53 signaling pathway, inhibits antiapoptotic genes, and triggers G1/S phase cell cycle stasis and programed cell death in GBM cells [16].

Currently, the influence of genes associated with programed cell death on the prognostic outcome of GBM is not fully understood, and systematic research is lacking. The complex microenvironment of GBM further increases the difficulty of treatment. Various cell types and molecular signaling pathways in the GBM microenvironment collectively promote tumor cell adaptation and progression [17–19]. Therefore, in‐depth research on the role of apoptosis‐related genes in GBM and their relationship with prognosis is crucial for revealing the pathogenesis of GBM, unearthing novel therapeutic targets, and enhancing patient prognostic outcomes. This study aims to explore the prognostic role of apoptosis‐related genes in GBM by integrating multiomics data, providing new insights into the clinical assessment and management of GBM.

## 2. Materials and Methods

### 2.1. Procurement and Manipulation of Transcriptome Information

RNA expression matrices and associated clinical information pertaining to GBM from the TCGA repository (*n* = 167) were utilized for the formulation of the model. The dataset from the CGGA database (*n* = 374) was used as a validation group for RNA‐seq data to ascertain the robustness and precision of the model. Any samples with incomplete information were removed, and if a gene appeared in multiple rows in the expression matrix, the data from those rows were averaged. Demographic and socioeconomic variables of clinical information were treated for the model adjustment. Data were converted to TPM format and log2‐transformed for subsequent analysis. Additionally, microarray data of GBM from the gliovis database (http://gliovis.bioinfo.cnio.es/), integrating Ducray, Freije, Gravendeel, Joo, LeeY, Murat, Phillips, and Rembrandt datasets, were used as a validation set, comprising a total of 825 samples. Six immunotherapy datasets were also utilized, including GBM_PRJNA482620 (*N* = 34), Melanoma_GSE100797 (*N* = 25), Melanoma_GSE78220 (*N* = 28), Melanoma_GSE91061 (*N* = 109), Melanoma_GSE106128 (*N* = 47), and RCC_Braun_2020 (*N* = 311). The normalizeBetweenArrays function from the limma package was utilized to standardize the microarray data. The Combat function from the SVA package was employed to mitigate batch effects across datasets.

### 2.2. Procurement and Manipulation of scRNA‐Seq Information

Single‐cell datasets were sourced from GEO repositories, comprising GSE182109 with 40 GBM neoplastic samples and GSE139448 with 3 GBM neoplastic samples. Data analysis was conducted utilizing R software (Version 4.1.3) in conjunction with the Seurat package. Quality control parameters for cells entailed mitochondrial content below 10%, hematopoietic cell content below 3%, and UMI counts and gene counts within the specified ranges of 200–20,000 and 200–6000, respectively [20]. Data normalization, identification of highly variable genes (2000 genes), and data transformation (mitigating the influence of the cell cycle with parameters vars‐to‐regress = c (“*S*‐score”, “G2M‐score”) were executed using the NormalizeData, FindVariableFeatures, and ScaleData functions from the Seurat package, respectively. Batch effect rectification was achieved using Harmony. Subsequently, the uniform manifold approximation and projection (UMAP) dimensionality reduction technique and the Louvain clustering algorithm, both from the Seurat package, were applied. Differential genes between clusters or cell types were determined using the FindAllMarkers function with parameters *p*‐value <0.05, log_2_ fold change (FC) >0.25, and expression proportion >0.1.

### 2.3. Cell Annotation and Enrichment Analysis

Markers for glioma cells (“GFAP,” “S100B,” “SOX2,” and “OLIG1”), oligodendrocyte cells (“MBP” and “OLIG2”), pericytes (“PDGFRB” and “ACTA2”), endothelial cells (“RAMP2,” “FLT1,” “CLDN5,” and “PECAM1”), T cells (“TRAC,” “CD3G,” “CD3E,” and “CD3D”), NK cells (“KLRD1”, “NCAM1,” “GNLY,” and “NKG7”), B cells (“IGHA2,” “IGHG3,” “IGHM,“and “CD79A”), myeloid cells (“FCGR3A,” “CD68,” “MARCO,” and “LYZ”), and mast cells (“MS4A2,” “GATA2,” and “KIT”) were utilized. Immune cell subsets were annotated using the sc‐type software [21].

The clusterProfiler R package facilitated the analysis and visualization of GO terms and KEGG pathways associated with the genes. To ensure the reliability and statistical significance of our results, we employed rigorous filtering criteria. Initially, we established a *p*‐value threshold, considering only enrichment results with a *p*‐value less than 0.05, to identify statistically significant enrichments of GO terms or KEGG pathways. Furthermore, we applied an additional filtering criterion using an adjusted *p*‐value <0.05 to account for multiple comparisons. By implementing these stringent filtering criteria, we effectively identified GO terms and KEGG pathways that are closely associated with the genes and statistically significantly enriched.

### 2.4. Acquisition of Prognostic Genes (Apoptosis Signature [AS])

Differential genes between tumor and adjacent tissues using default parameter settings (adjusted *p* value <0.05, |log_2_FC| > 1) were calculated using the limma software. Apoptosis‐related differential genes were filtered out, and a univariate Cox proportional hazards model was employed to discern prognostic genes (*p*‐value <0.05) from the dataset. These 63 prognostic genes were used to construct the AS model.

### 2.5. Establishment of Tumor‐Related Risk Signatures

Prognostic models were constructed utilizing 101 machine learning algorithms. Consequently, each patient was assigned a risk score based on this computational approach [22, 23]. The threshold for stratification was ascertained using the surv_cutpoint function, and patients in the TCGA cohort and other cohorts were categorized into high‐risk and low‐risk groups. Subsequent analysis focused on examining the predictive discrepancies between these groups and assessing the model’s precision.

### 2.6. Risk Signatures Generated by Machine Learning‐Based Ensemble Methods

To construct an AS model with high accuracy and robustness, we amalgamated 10 machine learning algorithms and 101 algorithmic combinations. The ensemble algorithms encompassed random survival forest (RSF), elastic net (Enet), lasso, ridge, stepwise Cox, CoxBoost, Cox partial least squares regression (plsRcox), supervised principal component (SuperPC), generalized boosted regression model (GBM), and survival support vector machine (survival‐SVM). The process for generating the signature was as follows: (a) univariate Cox regression analysis was conducted to identify prognostic genes in three datasets, including TCGA cohort (as detailed in the preceding step); (b) subsequently, 101 algorithm combinations were applied to the prognostic genes to construct a predictive model based on the leave‐one‐out cross‐validation (LOOCV) framework within the TCGA‐GBM cohort; (c) all models were subjected to validation in the validation datasets; (d) for each model, the Harrell concordance index (*C*‐index) was computed across all validation datasets, and the model with the highest average *C*‐index was deemed optimal. The established model was adjusted for demographic and socioeconomic variables, including age, sex, race and ethnicity, educational level, family income, and marital status.

### 2.7. Examination of Cellular Interactions

The likelihood of intercellular communication was evaluated utilizing the CellChat package V.1.6.114. The normalized gene expression matrix was integrated to quantitatively infer and analyze intercellular communication networks. To determine communications specific to cell states, we initially pinpointed signaling genes with differential expression across all cell groups in the scRNA‐seq dataset, employing the Wilcoxon rank sum test at a 0.05 significance level. The identifyOSAerExpressedGenes, identifyOSAerExpressedInteraction, and ProjectData functions were subsequently applied with standard parameters to prepare the data. The computeCommunProb, filterCommunication, and computeCommunProbPathway functions were then utilized to discern potential ligand‐receptor interactions. Ultimately, the aggregateNet function was employed to construct the cellular communication network.

### 2.8. Genomic Alteration Examination

GISTIC analysis was adopted to identify the genomic event enrichment (https://gatkforums.broadinstitute.org). Copy number alterations were scrutinized using GISTIC 2.0 software, and the tumor mutation load was quantified for each patient utilizing the maftools software.

### 2.9. Association Study Between Prognostic Model and Tumor Immunity

The extent of immune cell infiltration for each patient from the TCGA repository was ascertained with the IOBR software, which integrates findings from seven distinct assessment methodologies. Heatmaps were generated from this data to delineate the relative abundance of immune cell infiltration within the tumor microenvironment (TME).

### 2.10. Chemotherapeutic Susceptibility

The half‐maximal effective concentration (IC50) and area under the dose–response curve (AUC) for prevalent chemotherapeutic agents were computed using the R package “oncoPredict” in conjunction with drug databases GSDCv2 and CTRP. This facilitated the evaluation of the correlation between risk scores and drug responsiveness. The IC50 or AUC values across the two risk categories were juxtaposed using the Wilcoxon rank‐sum test.

### 2.11. Immunotherapy Response Forecasting

The tumor immune dysfunction and exclusion (TIDE) algorithm [24–26] (harvard.edu) was employed to predict immune responses within the TCGA dataset, assessing the AS variance in immunotherapy responsiveness.

### 2.12. Statistical Analysis

All data manipulation, statistical analysis, and graphical representation were executed using R 4.1.3 software. The relationship between two continuous variables was gauged using Pearson’s correlation coefficient. Categorical variables were contrasted using the chi‐square test, and continuous variables were compared using the Wilcoxon rank‐sum test or *t*‐test. The optimal threshold was identified using the survminer package. Cox regression and Kaplan–Meier analysis were conducted using the survival package.

## 3. Results

### 3.1. Identification of Model‐Building Genes

As shown in Figure 1A, there was a notable divergence in gene expression levels between neoplastic and neighboring tissues, with genes such as BRCA1, CHEK2, and IKBKE substantially amplified in tumor tissues, while genes such as ZMYND11, MAPK8, and RPS3 exhibited notable elevation in adjacent nontumor tissues. As illustrated in Figure 1B, we screened for genes intimately linked to the prognostic outcome of GBM and found that BRCA1, CHEK2, IKBEKE, and PPIA constituted adverse indicators for the prognostic trajectory of GBM patients, whereas ZMYND11, MAPK8, RPS3, and GHITM were protective factors. The top 10 risk genes with the highest hazard ratios (HRs) and the top six protective genes with the lowest HRs are detailed in Table 1. As shown in Figure 1C, the most relevant GO biological processes (GOBPs) were regulation of apoptotic signaling pathway, intrinsic apoptotic signaling pathway, extrinsic apoptotic signaling pathway, and negative regulation of apoptotic signaling pathway. The GO cellular components (GOCCs) were the membrane raft, the membrane microdomain, and the focal adhesion. The GO molecular functions (GOMFs) were ubiquitin‐like protein ligase binding, ubiquitin protein ligase binding, and protein serine/threonine kinase activity. The KEGG results suggested that these genes were enriched in pathways such as Epstein–Barr virus infection, human cytomegalovirus infection, apoptosis, microRNAs in cancer, HIV‐1 infection, hepatitis B, and necroptosis (Figure 1D). Figure 1E illustrates the manifestation patterns of these genes across diverse autosomal chromosomes. The PCA plot after batch effect removal for each dataset is shown in Figure 1F. Furthermore, we observed amplifications of these prognosis‐related genes on chromosomes 5–9, 16, and 20, while deletions were noted on chromosomes 2, 10, 15, and 22 (Figure 1G).

Figure 1Displays the outcomes of model‐building gene identification. (A) Heatmap depicting the divergent expression of apoptosis‐associated genes between GBM and surrounding non‐neoplastic tissues. (B) Association study between apoptosis‐associated genes and GBM prognostic outcomes. (C, D) Bar charts illustrating the results of GO and KEGG enrichment analyses for differentially expressed genes. (E) Circos diagram illustrating the connection between apoptosis‐associated genes and GBM prognostic outcomes. (F) PCA diagram of each dataset following the elimination of batch effects. (G) Bar chart displaying the CNV of prognostic genes.

**Figure:**
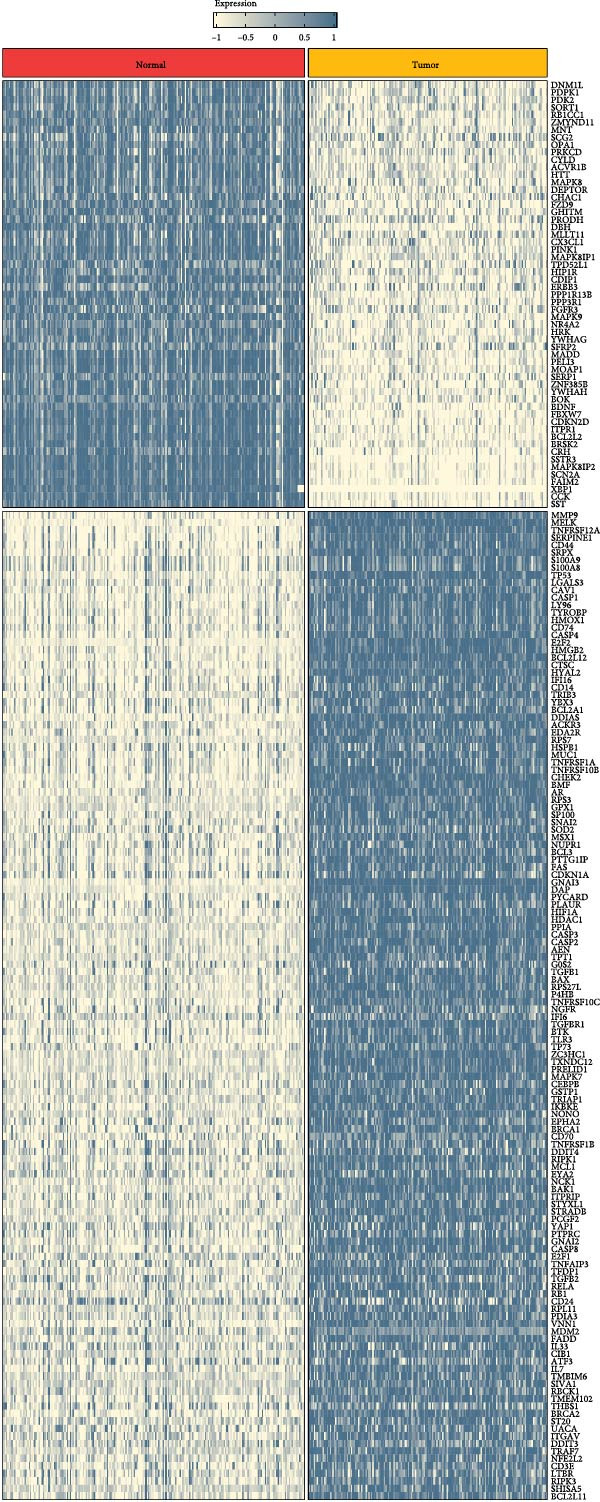
(A)

**Figure:**
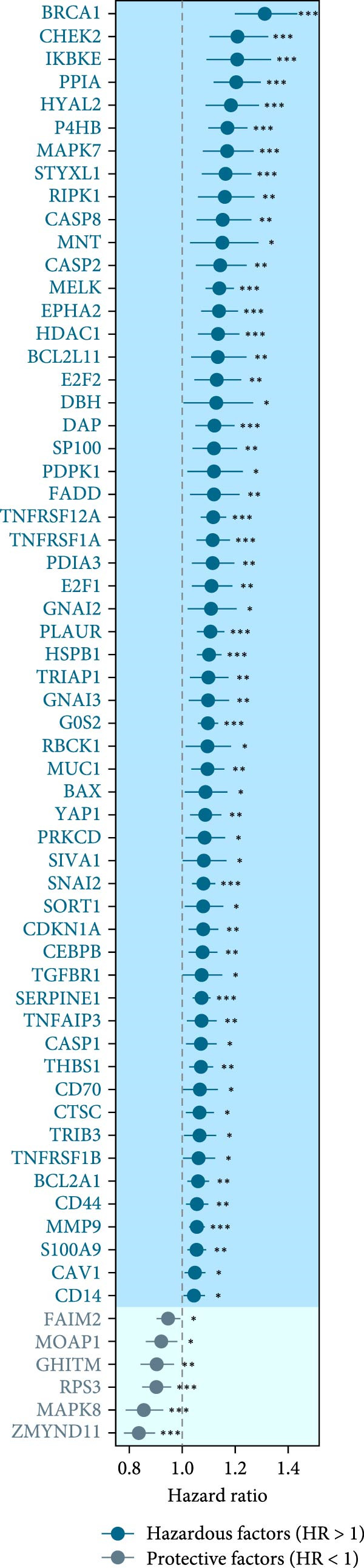
(B)

**Figure:**
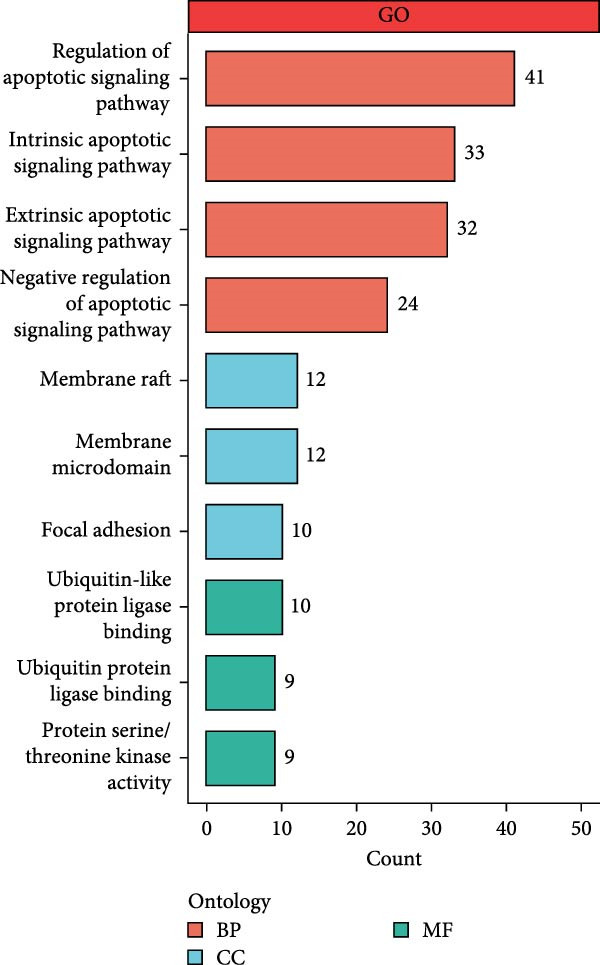
(C)

**Figure:**
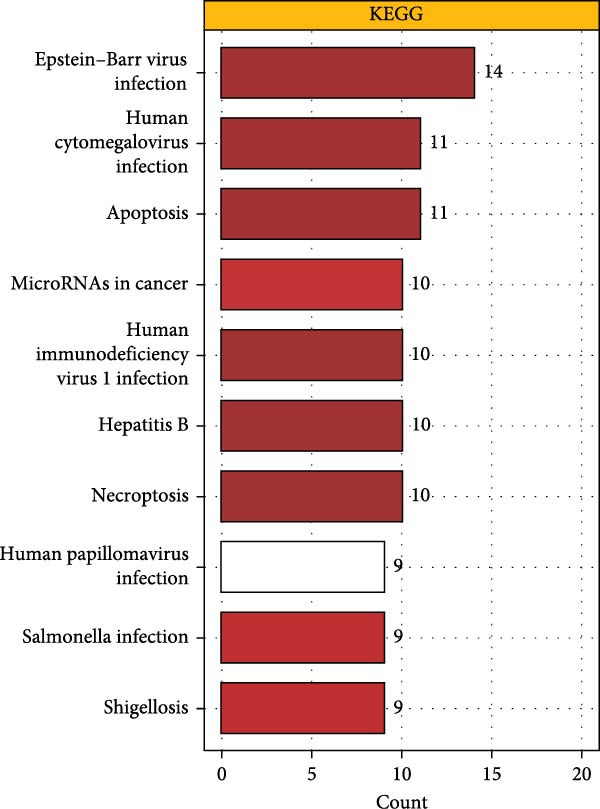
(D)

**Figure:**
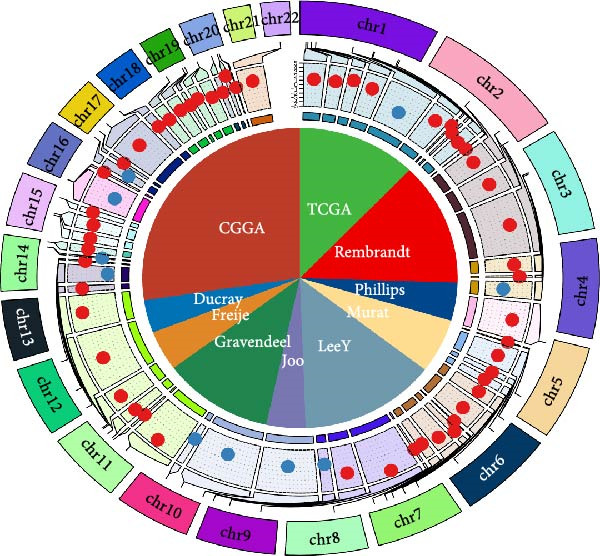
(E)

**Figure:**
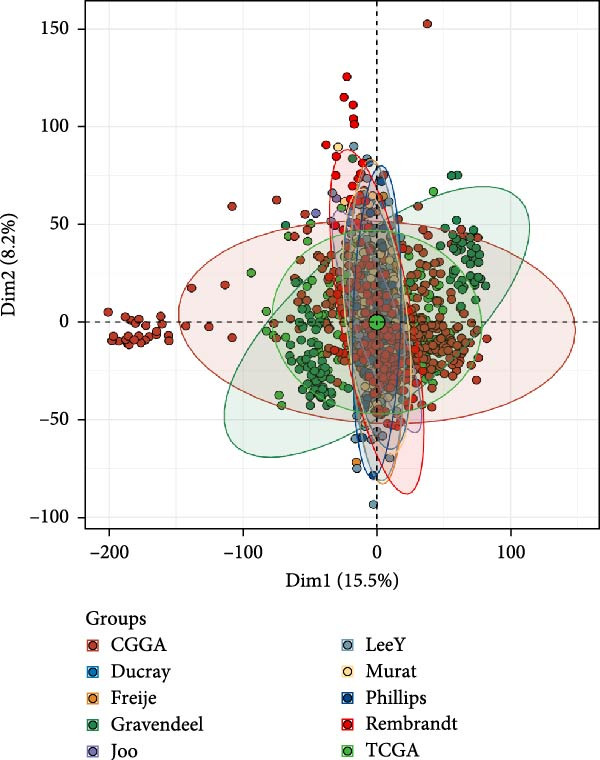
(F)

**Figure:**
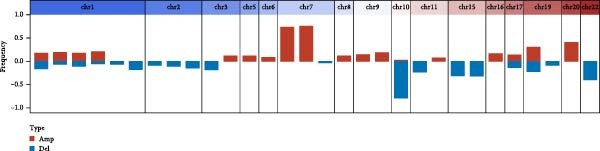
(G)

**Table 1:** Association study linking genes associated with programed cell death and the prognostic outcome of GBM.

Gene	Coef	se	*z*	*p*	HR	95% CI
BRCA1	0.27	0.05	5.88	<0.001	1.31	1.20–1.43
CHEK2	0.19	0.05	4.05	<0.001	1.21	1.10–1.32
IKBKE	0.19	0.05	3.66	<0.001	1.21	1.09–1.34
PPIA	0.19	0.04	4.89	<0.001	1.20	1.12–1.30
HYAL2	0.17	0.04	3.90	<0.001	1.18	1.09–1.29
P4HB	0.16	0.03	4.84	<0.001	1.17	1.10–1.25
MAPK7	0.16	0.04	3.72	<0.001	1.17	1.08–1.27
STYXL1	0.15	0.04	3.69	<0.001	1.16	1.07–1.26
RIPK1	0.15	0.05	3.18	<0.001	1.16	1.06–1.27
CASP8	0.14	0.05	3.11	<0.001	1.15	1.05–1.26
Protective effect gene in GBM						
ZMYND11	0.18	0.04	5.04	<0.001	0.84	0.78–0.90
MAPK8	0.16	0.04	3.70	<0.001	0.85	0.79–0.93
RPS3	0.10	0.03	3.36	<0.001	0.90	0.85–0.96
GHITM	0.10	0.04	2.86	<0.001	0.90	0.84–0.97
MOAP1	0.08	0.03	2.52	0.01	0.92	0.86–0.98
FAIM2	0.05	0.02	2.26	0.02	0.95	0.90–0.99

### 3.2. Construction of the AS Model

We employed 101 algorithms to model the aforementioned genes. Due to the minimal difference in average *C*‐index among the top 8 models (at the thousandths place), and considering that the *C*‐index of several top‐ranked models was relatively low (less than 0.5) in certain datasets, we comprehensively selected the CoxBoost + ridge model as the optimal model (Figure 2A). The PCA plots before and after batch effect removal for TCGA and GTEx data are shown in Supporting Information 1: Figure S1A. We selected RSP3, HSPB1, STYXL1, G0S2, and PLAUR genes for modeling. The expression heatmap of each gene in the high‐ and low‐risk groups is shown in Supporting Information 1: Figure S1B. Subsequently, we used the AS model to calculate survival analysis results for GBM in various datasets. It was noted that individuals classified in the AS high‐risk category exhibited inferior survival prospects relative to those in the AS low‐risk category across various datasets(Figure 2B–G). As shown in Figure 2H, in the CCGA dataset (*N* = 374), the mortality rate among patients in the AS high‐risk cohort was markedly higher compared to the AS low‐risk cohort (*p*  < 0.001). In a parallel manner, within the TCGA dataset (*N* = 167) and Frejie + Joo (*N* = 114) database, the mortality rate for patients in the AS high‐risk cohort was significantly elevated relative to the AS low‐risk cohort (*p*  < 0.001) (Figure 2I,J). Survival analysis results of the model in the immunotherapy dataset (Figure 2K–P). Ultimately, we employed the IPS value from the TCIA repository to assess disparities in immunotherapy among the various groups (Figure 2Q–T). Notable distinctions were evident between the AS high‐risk and low‐risk groups in instances of CTLA4 and PD‐L1 negativity (*p* = 0.0039).

Figure 2Presents the results of AS model construction. (A) Heatmap of *C*‐index for 101 algorithms. (B–J) Survival analysis results for GBM datasets. (K–P) Survival analysis results for immunotherapy datasets. (Q–T) Violin plots of IPS differences between high and low AS cohorts.

**Figure:**
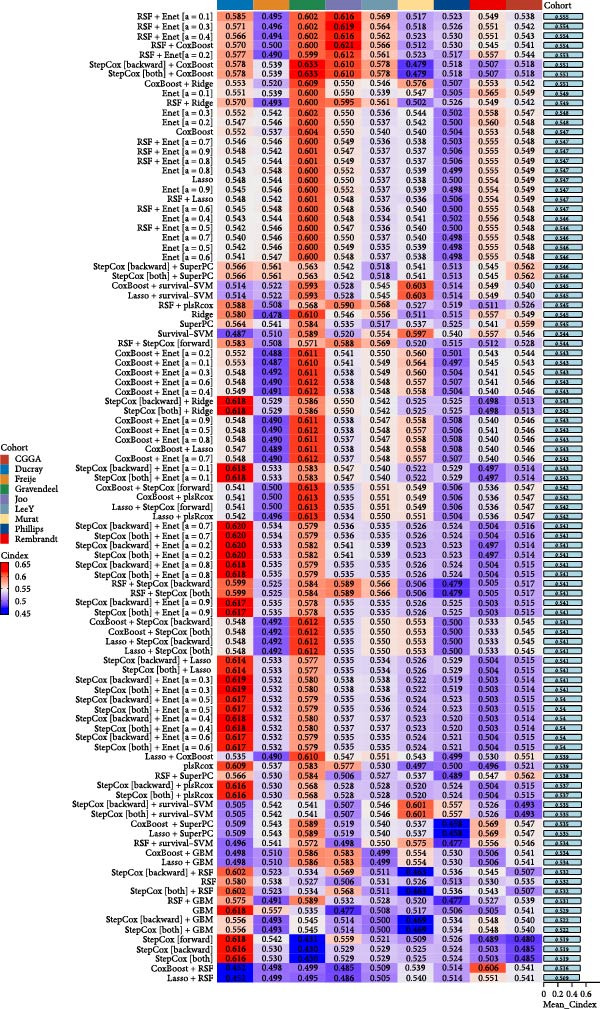
(A)

**Figure:**
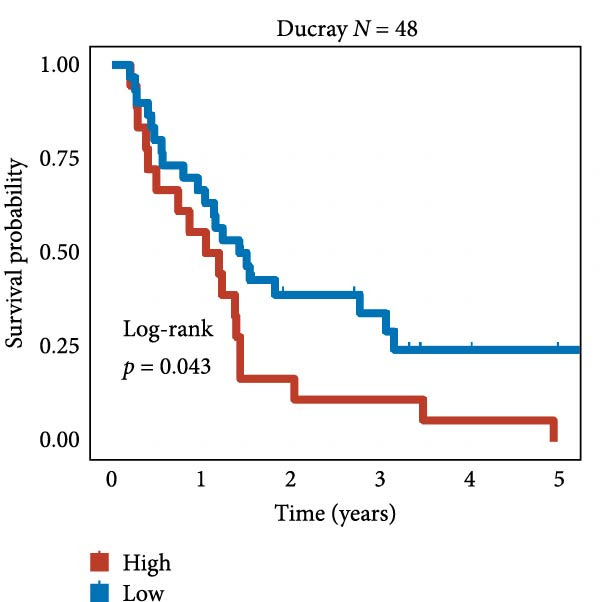
(B)

**Figure:**
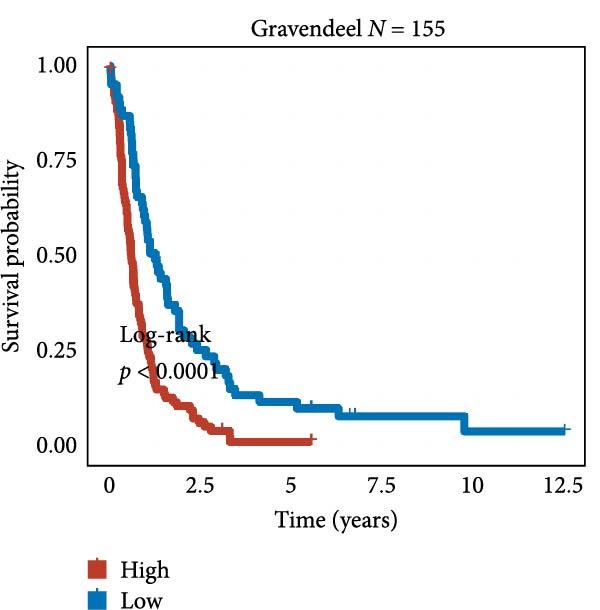
(C)

**Figure:**
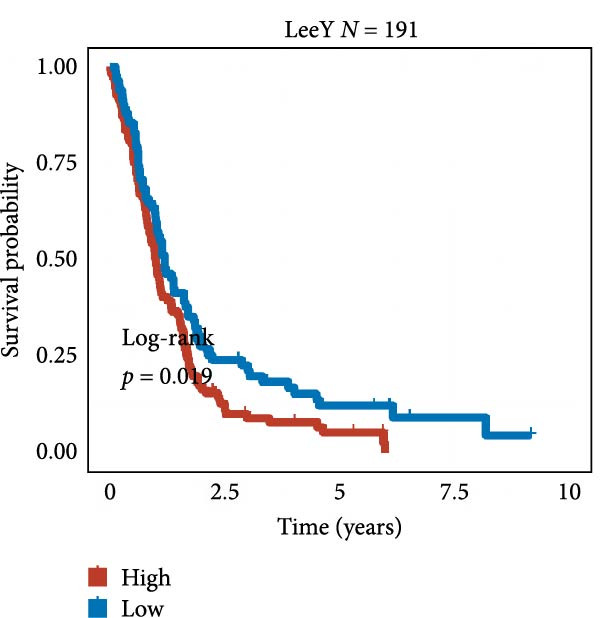
(D)

**Figure:**
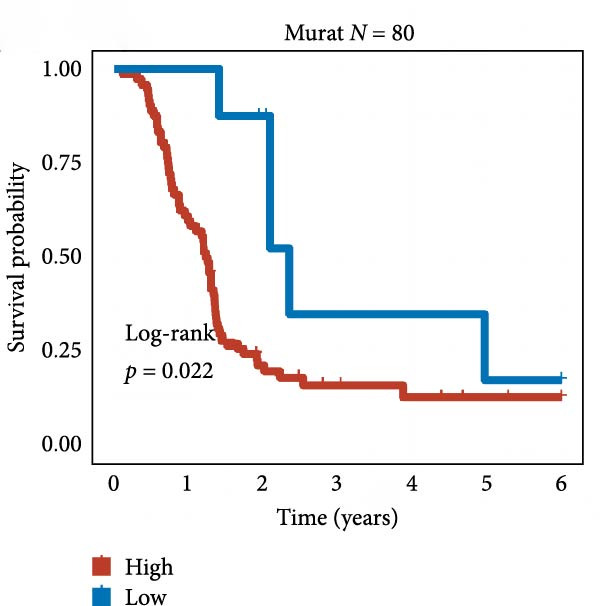
(E)

**Figure:**
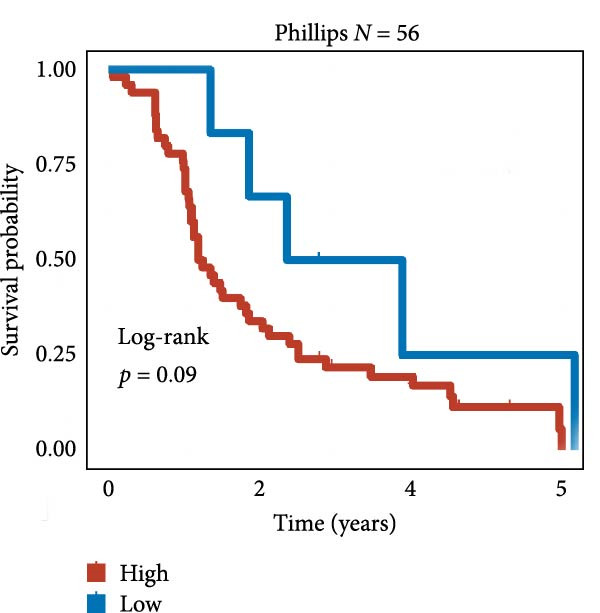
(F)

**Figure:**
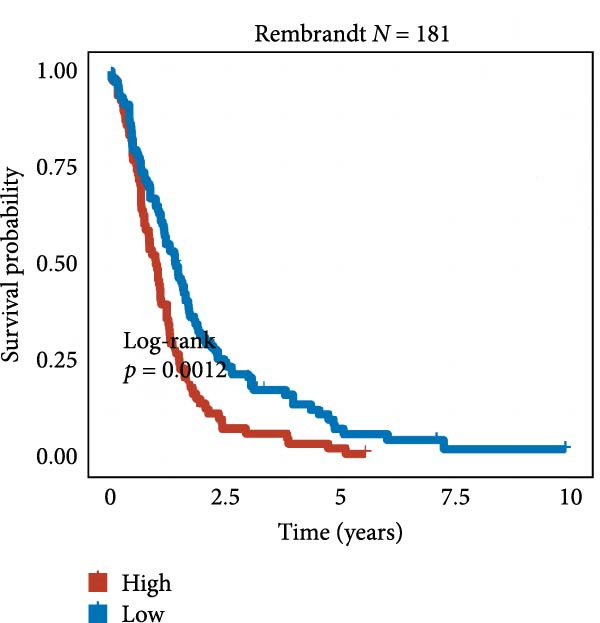
(G)

**Figure:**
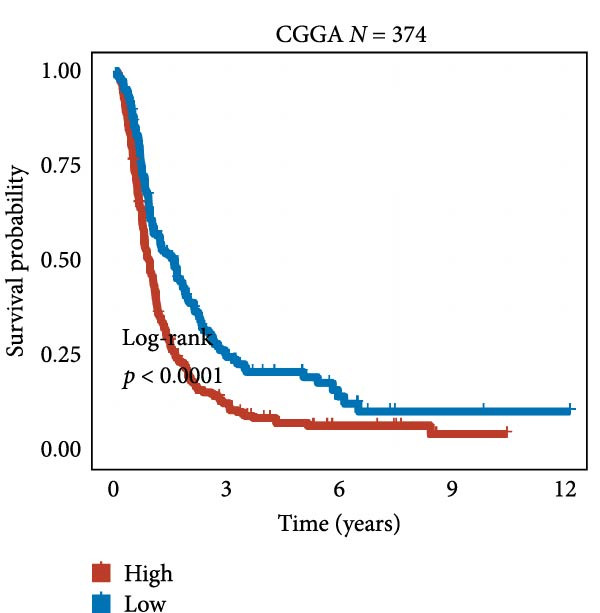
(H)

**Figure:**
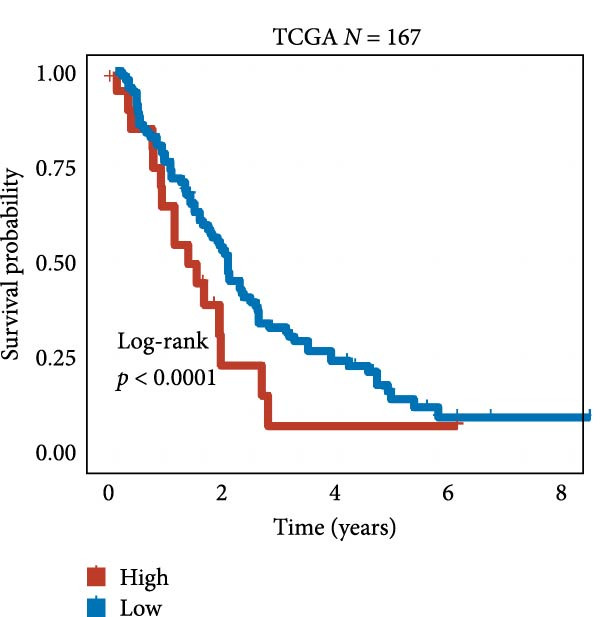
(I)

**Figure:**
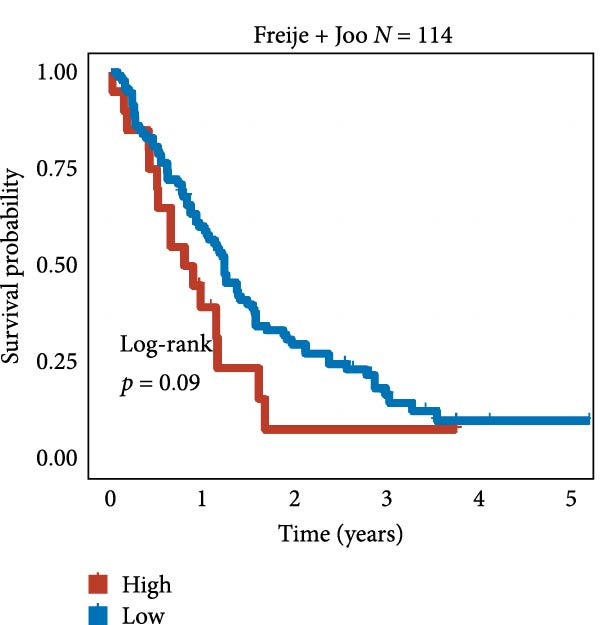
(J)

**Figure:**
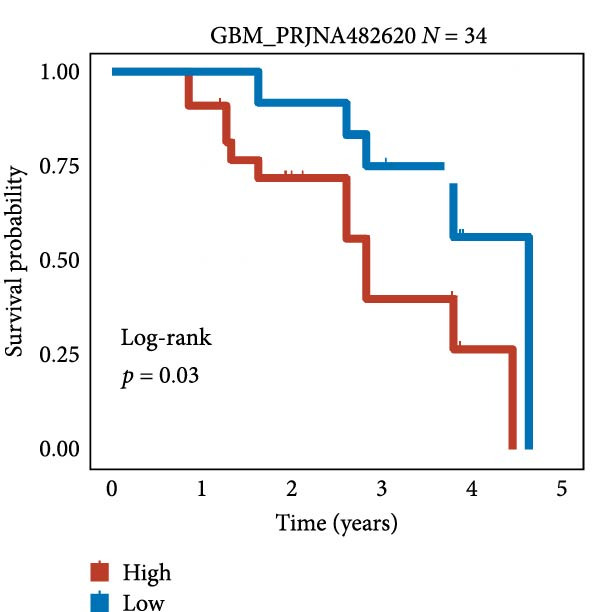
(K)

**Figure:**
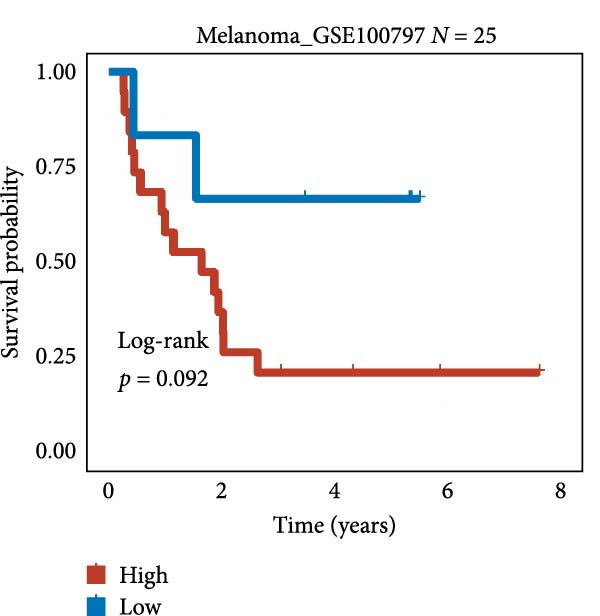
(L)

**Figure:**
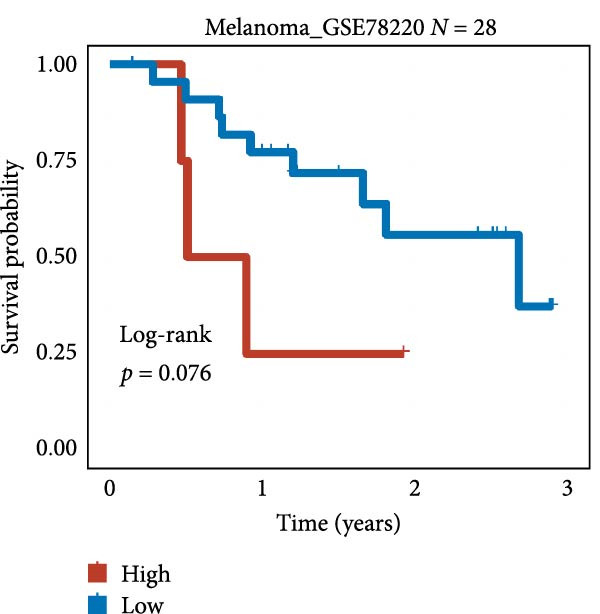
(M)

**Figure:**
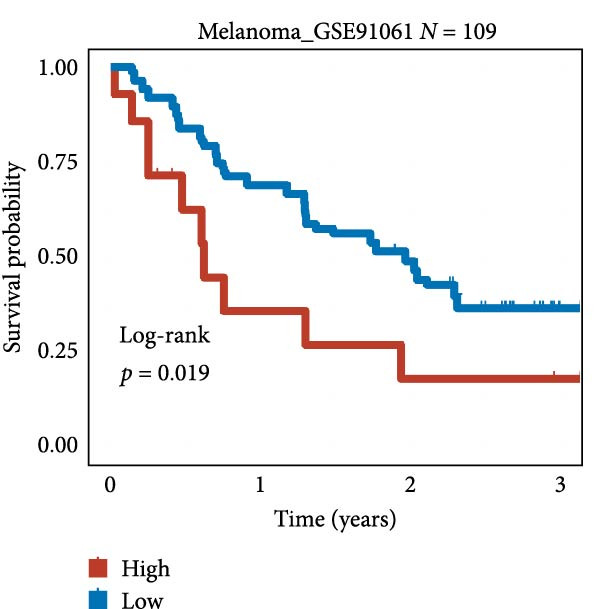
(N)

**Figure:**
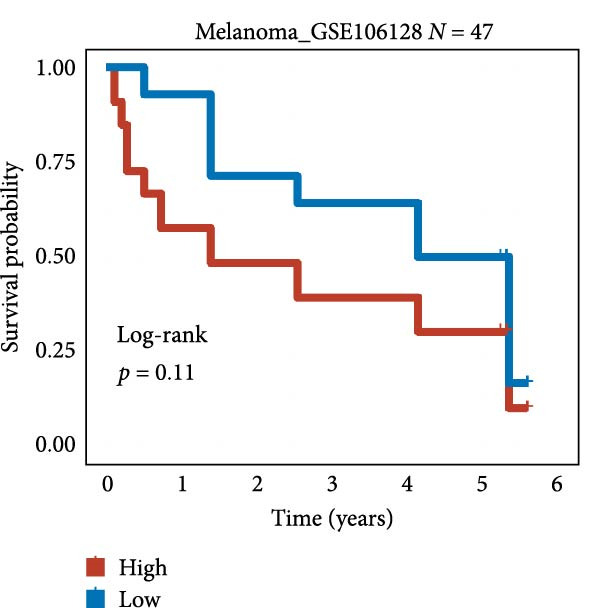
(O)

**Figure:**
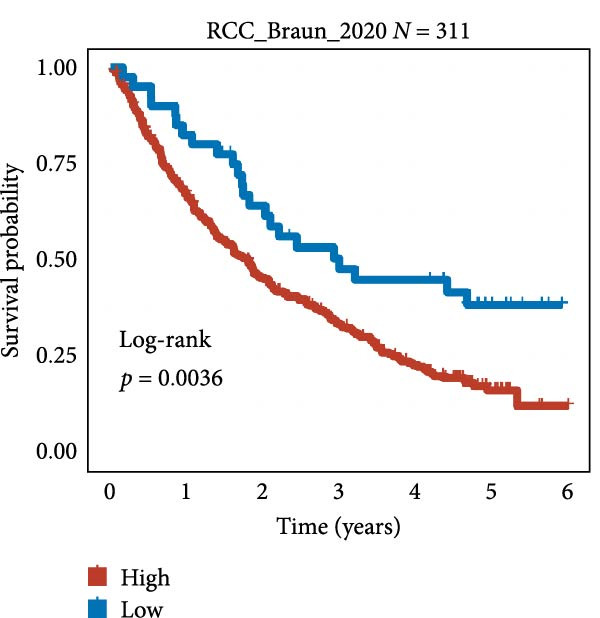
(P)

**Figure:**
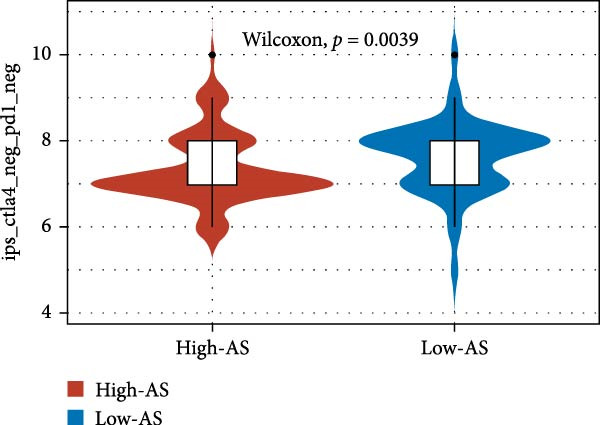
(Q)

**Figure:**
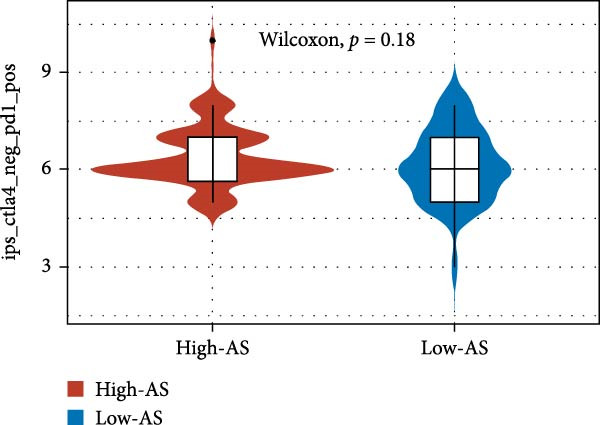
(R)

**Figure:**
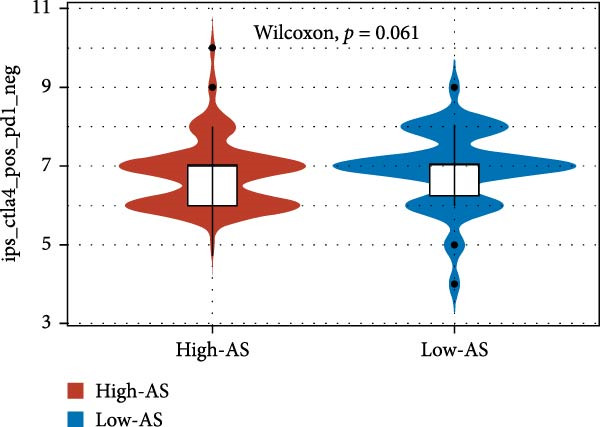
(S)

**Figure:**
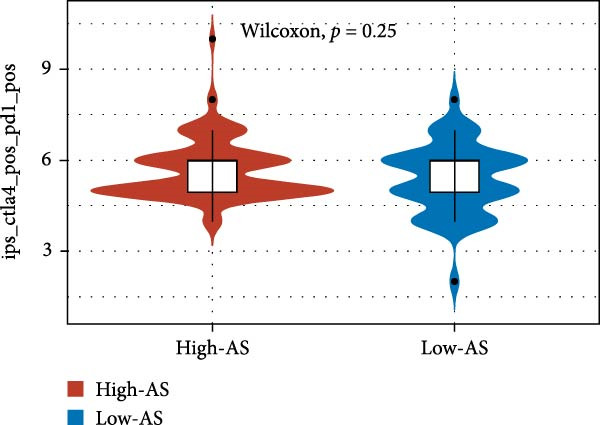
(T)

### 3.3. Model Comparison

We compared the *C*‐INDEX of the AS model with age and gender for prognosis in various datasets. It was found that in the TCGA and CGGA datasets, the AS model had a higher *C*‐INDEX than age and gender for predicting GBM prognosis (Figure 3A). Figure 3B shows the PCA plots for various GBM datasets. We visualized the prognostic value of the AS model for GBM using timeROC. It was observed that the AS model exhibited good predictive value in datasets such as Ducray, Gravendeel, CGGA, and TCGA, particularly in the TCGA dataset, where AUC at 1 Year: 0.70, AUC at 2 Years: 0.73, and AUC at 3 Years: 0.73 (Figure 3C). Subsequently, we compared the *C*‐index value of our model with 30 other models reported in the literature. As shown in Figure 3D, the AS model’s *C*‐index was generally higher than that of most other prediction models.

Figure 3The outcomes of model comparison. (A) Bar chart juxtaposing the *C*‐index of the AS model against age and gender across multiple datasets. (B) PCA diagrams for various GBM datasets. (C) TimeROC findings for various GBM datasets. (D) Comparative analysis of *C*‐index values between the AS model and 30 other models documented in the literature across multiple datasets.

**Figure:**
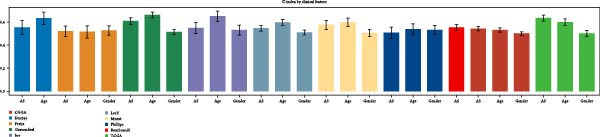
(A)

**Figure:**
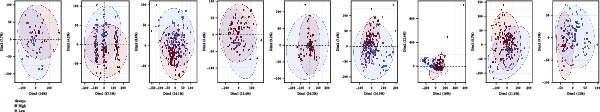
(B)

**Figure:**
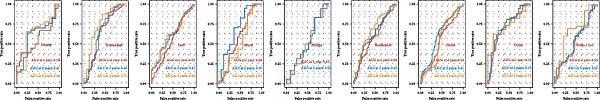
(C)

**Figure:**
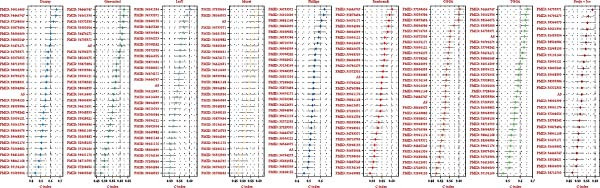
(D)

### 3.4. Tumor Immune Analysis

We predicted tumor immunity using the CIBERSORT and TIDE algorithms. As shown in Figure 4A, a marked divergence was observed in the levels of plasma cells between the AS high and low groups (*p* = 0.00046). There was no significant difference in monocytes(Figure 4B) and CD8 (Figure 4E)between the two groups. As depicted in Figure 4C, a notable disparity was evident in the presence of tumor‐associated macrophages (TAMs) between the AS high and low groups (*p* = 5.7e − 10). Such differences were also observed in MDSCs (Figure 4D) and IFNG (Figure 4F). The heat map shows the connection between AS and the expression of immune‐related genes (Figure 4G). The radar chart illustrating the variations in the association between AS and immune‐related pathways highlighted substantial differences in the activation of these pathways between the AS high and low groups, especially concerning checkpoint molecules, antitumor cytokines, Th1 signature, and cancer‐associated fibroblasts (Figure 4H). Subsequently, we assessed the disparities between the AS high and low cohorts using seven software tools, including TIMER, CIBERSORT, CIBERSORT‐ABS, MCPcounter, EPIC, xCell, and TISIDB. The heatmap findings are depicted in Figure 4I. We examined the histological sections of neoplastic tissues from patients in the AS high‐risk and low‐risk categories within the TCGA repository and found that tumor tissues from AS high‐risk patients exhibited poorer differentiation, suggesting poor prognosis (Figure 4J).

Figure 4Displays the outcomes of tumor immunity analysis. (A–F) Violin diagrams illustrating the disparities in tumor immunity between the AS high and low groups as predicted by CIBERSORT and TIDE algorithms. (G) Association findings between AS and expression of genes related to immunity. (H) Radar chart depicting the differences in AS and pathways associated with immunity. (I) Heatmap showing the variations in immune infiltration assessed by seven software tools between the AS high and low groups. (J) Histological section images of samples from the AS high and low groups.

**Figure:**
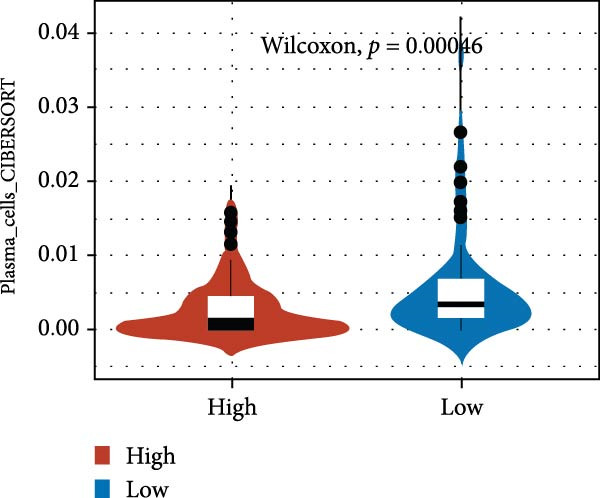
(A)

**Figure:**
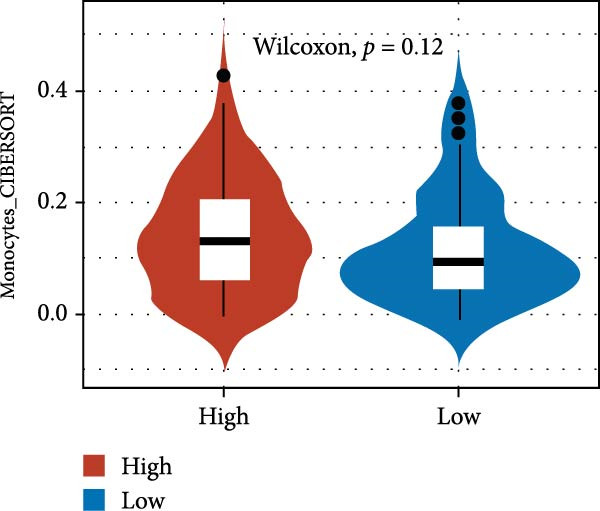
(B)

**Figure:**
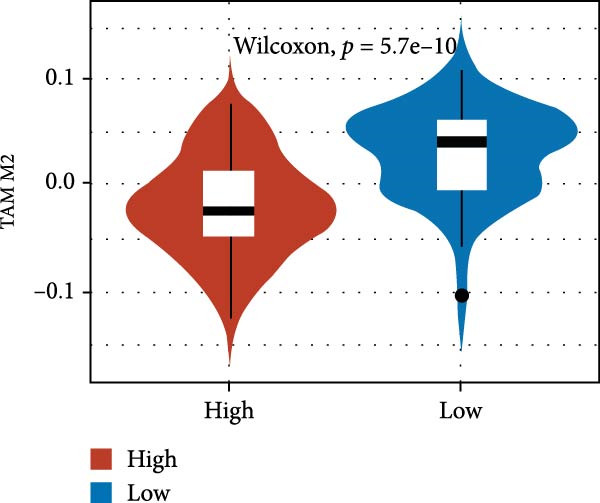
(C)

**Figure:**
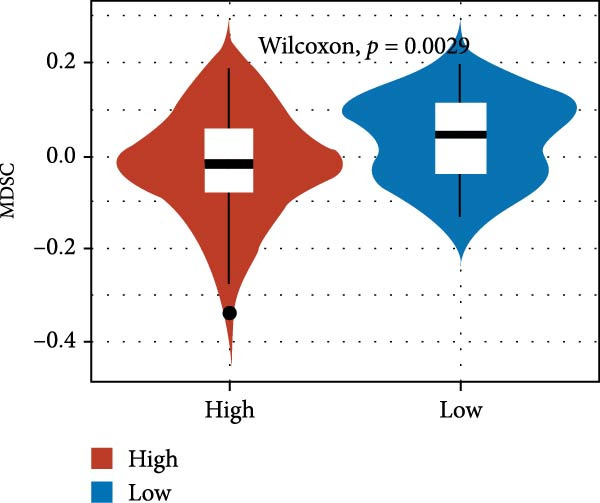
(D)

**Figure:**
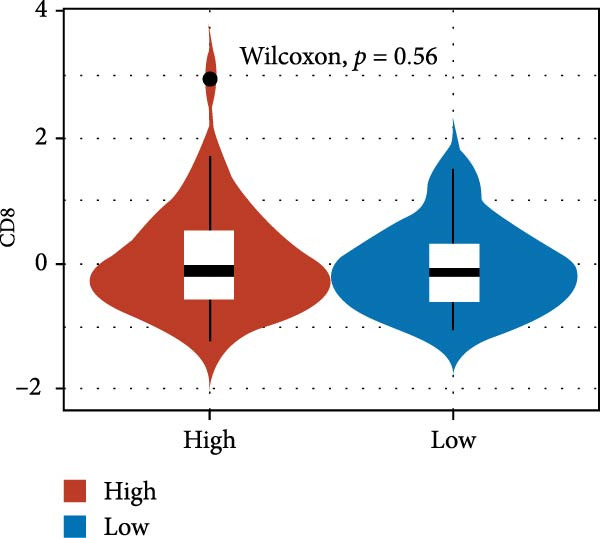
(E)

**Figure:**
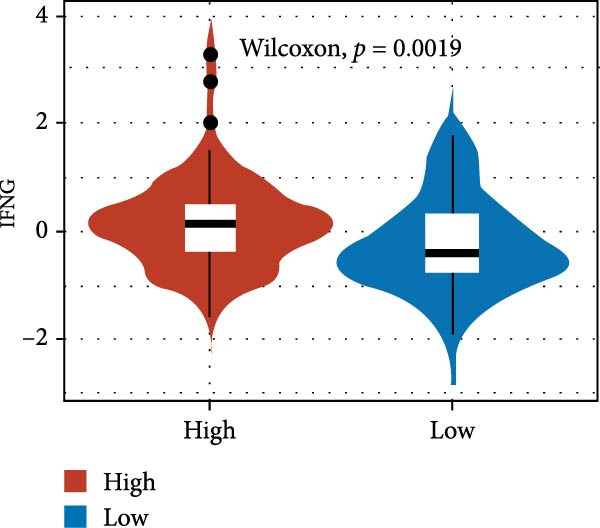
(F)

**Figure:**
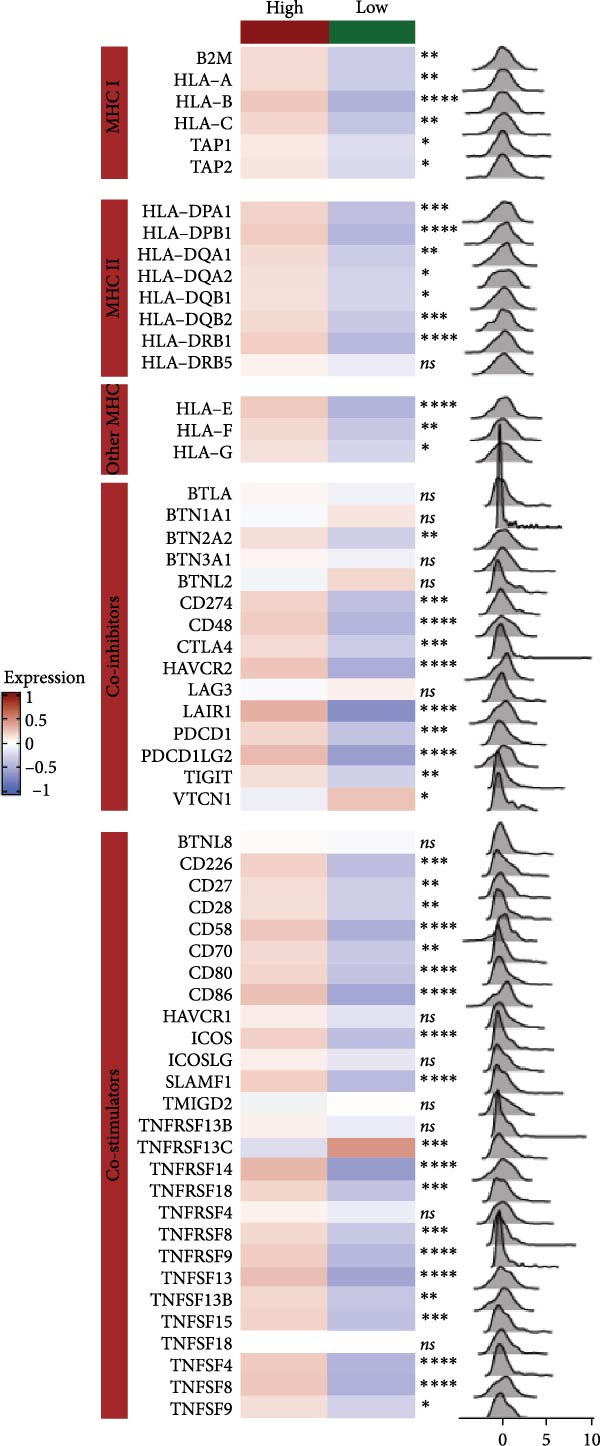
(G)

**Figure:**
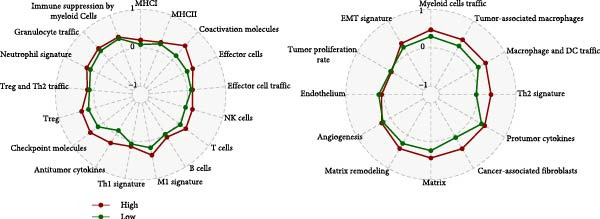
(H)

**Figure:**
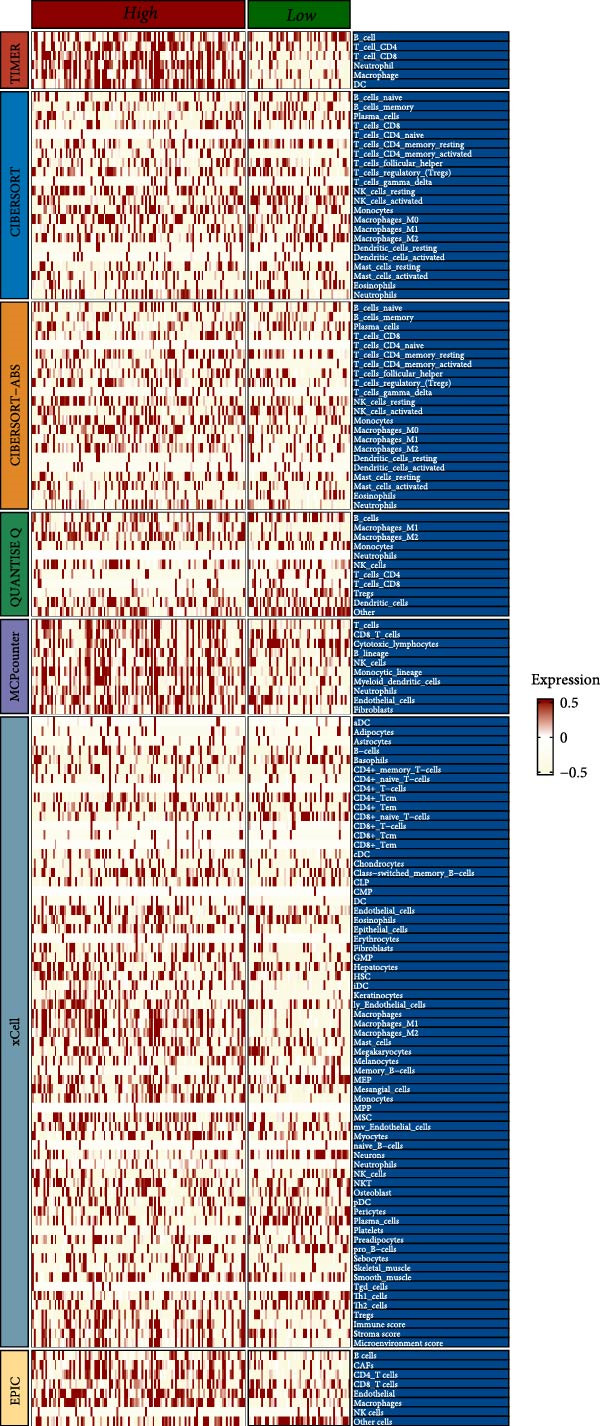
(I)

**Figure:**
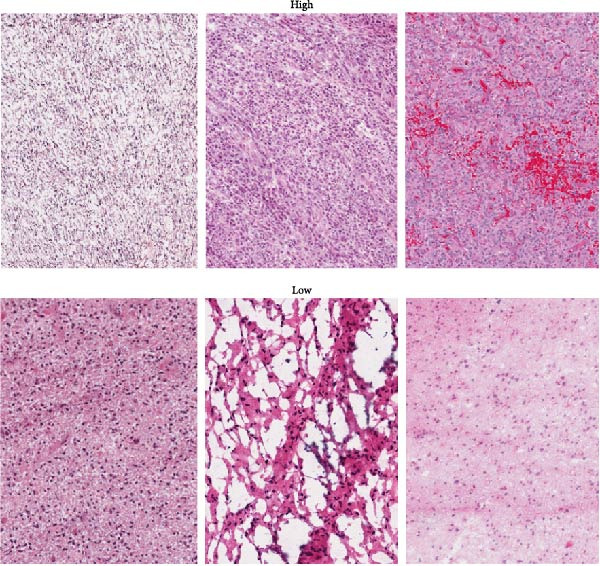
(J)

Moreover, we conducted a functional analysis of the Msigdb gene set for the AS high‐ and low‐risk cohorts. As illustrated in Supporting Information 2: Figure S2A, there were notable differences in enrichment between the AS high‐ and low‐risk cohorts. KEGG analysis highlighted significant disparities in the pentose phosphate pathway, KEGG galactose metabolism, and KEGG JAK‐STAT signaling pathway between the AS high‐ and low‐risk cohorts (Supporting Information 2: Figure S2B–D). In a parallel manner, GO results revealed significant differences in GPBP_glucose_6_phosphate metabolic process, GOBP T cell extravasation, and mammary gland involution between the AS high‐ and low‐risk cohorts (Supporting Information 2: Figure S2E–G). Supporting Information 2: Figure S2H presents the correlation analysis between AS and TIP tumor immunity and immunotherapy pathways.

Furthermore, we employed gistic2.0 to graphically represent the CNV segment data from TCGA for the high and low AS cohorts (Supporting Information 3: Figure S3A,B). We calculated mutation results based on TCGA mutation data (Supporting Information 3: Figure S3C) and found no discernible CNV disparities between the AS high‐ and low‐risk cohorts (Supporting Information 3: Figure S3D). We also calculated TMB values based on TCGA mutation data and combined them with AS groups to obtain four prognostic groups. It was observed that in cases with the same TMB burden, individuals in the AS high‐risk category exhibited markedly reduced survival probabilities compared to those in the AS low‐risk category (*p* = 0.00074) (Supporting Information 3: Figure S3E).

### 3.5. AS Analysis of Single‐Cell Data

Subsequently, we analyzed the GSE182109 single‐cell sequencing dataset, which contained a total of 192,503 cells, including 71,967 glioma cells, 4595 oligo cells, 26,088 NK/T cells, 55,181 macrophages, 2451 pericytes, 24,208 mDCs, 5565 monocytes, 1348 endothelial cells, and 1100 B cells (Figure 5A). The risk score results are shown in Figure 5B. Figure 5C presents the composition of various cell types in the GSE182109 single‐cell dataset, with glioma cells and macrophages being the most abundant.

Figure 5Illustrates the findings from the AS analysis of single‐cell data. (A–C) Analysis outcomes for the GSE182109 dataset. (D–F) Analysis outcomes for the GSE139448 dataset. (G–H) Violin diagrams of AS scores for the GSE182109 and GSE139448 datasets. (I–M) Findings on the disparities in cellular communication between the high and low AS groups.

**Figure:**
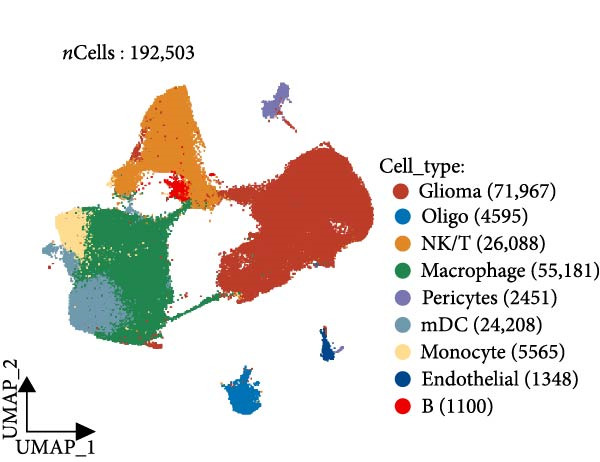
(A)

**Figure:**
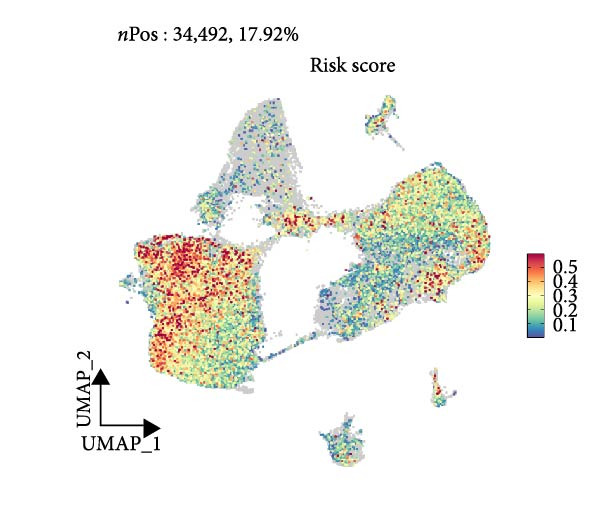
(B)

**Figure:**
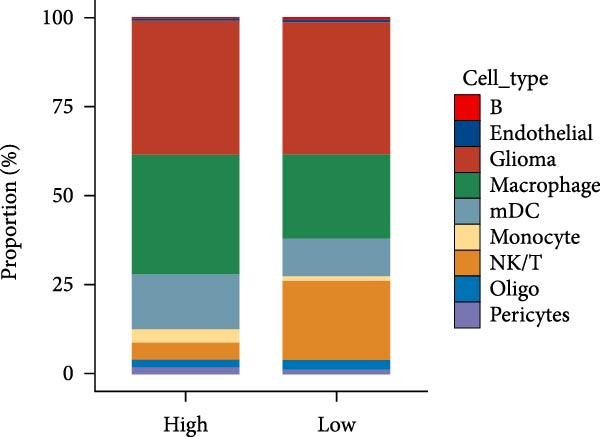
(C)

**Figure:**
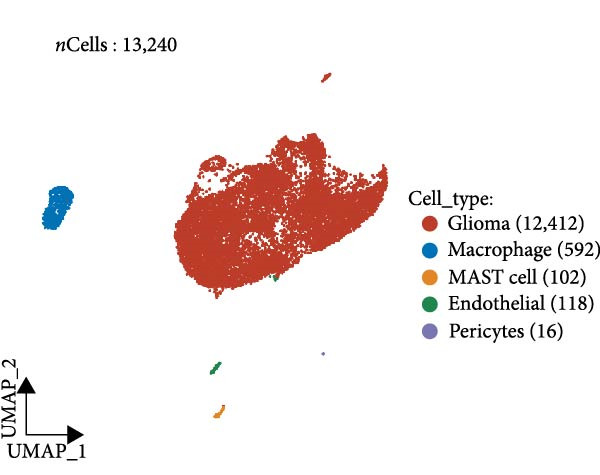
(D)

**Figure:**
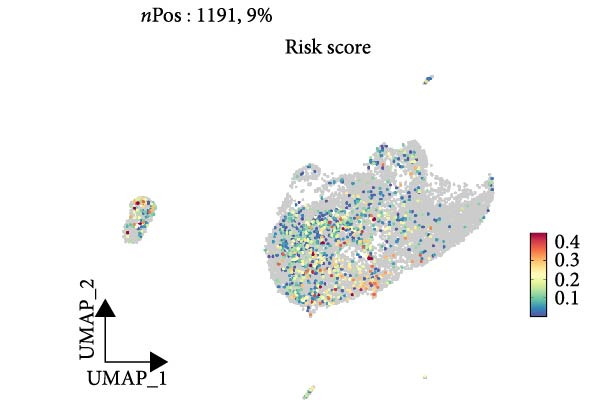
(E)

**Figure:**
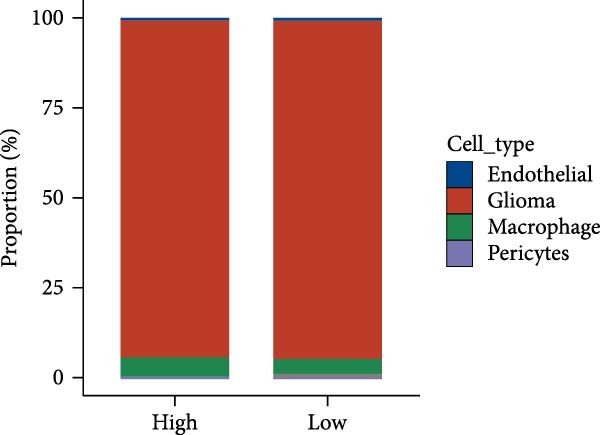
(F)

**Figure:**
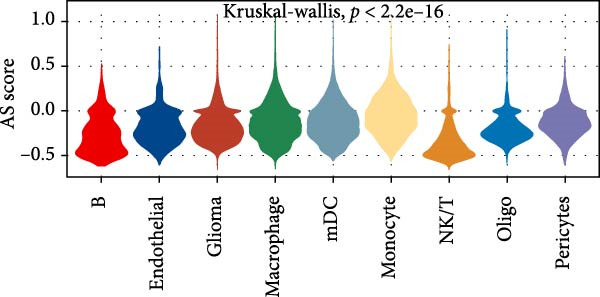
(G)

**Figure:**
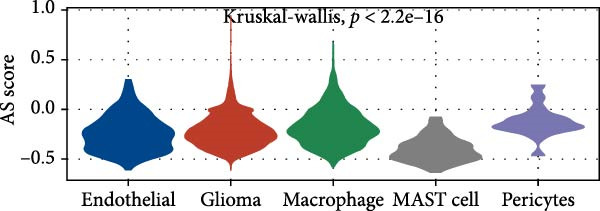
(H)

**Figure:**
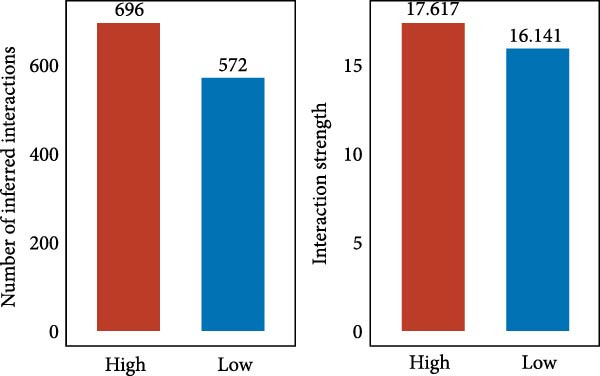
(I)

**Figure:**
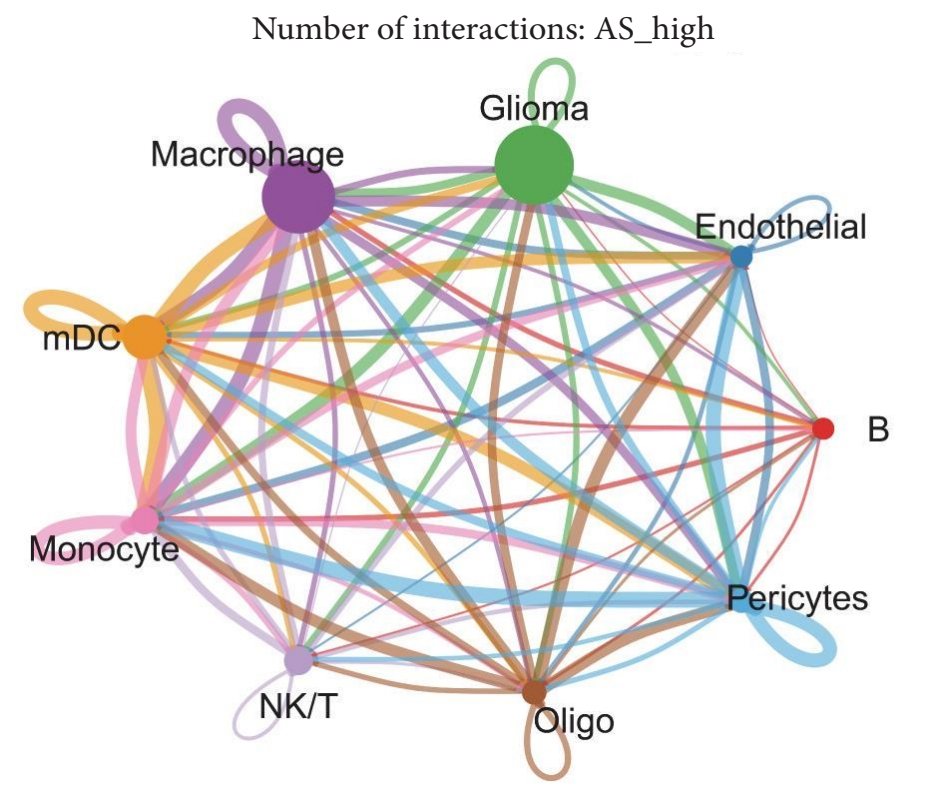
(J)

**Figure:**
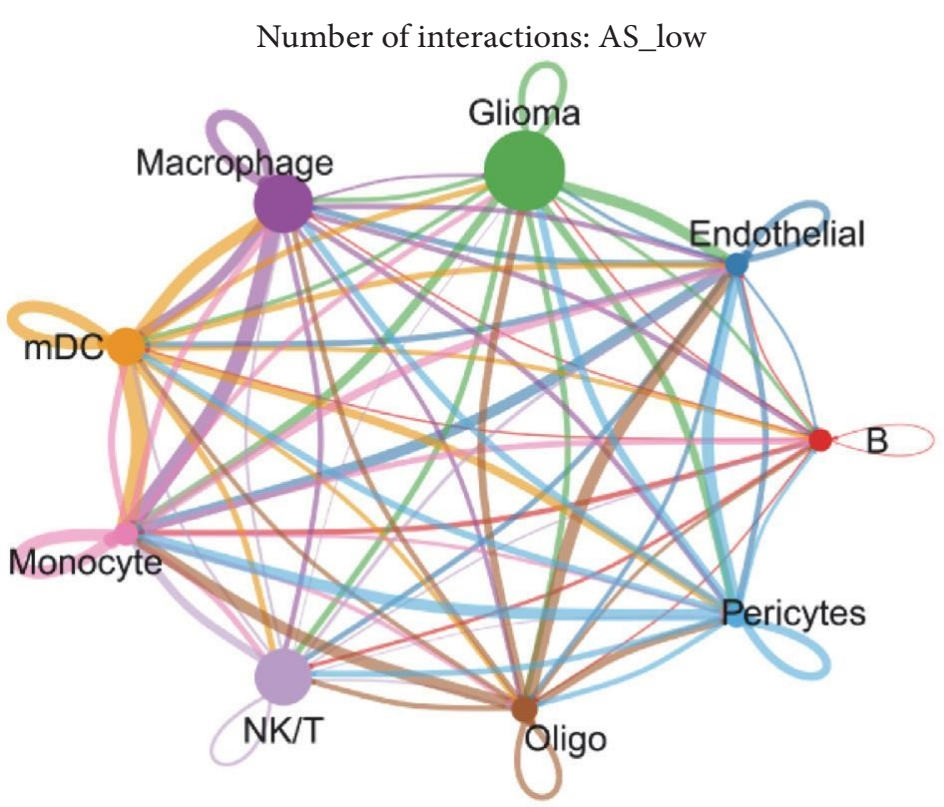
(K)

**Figure:**
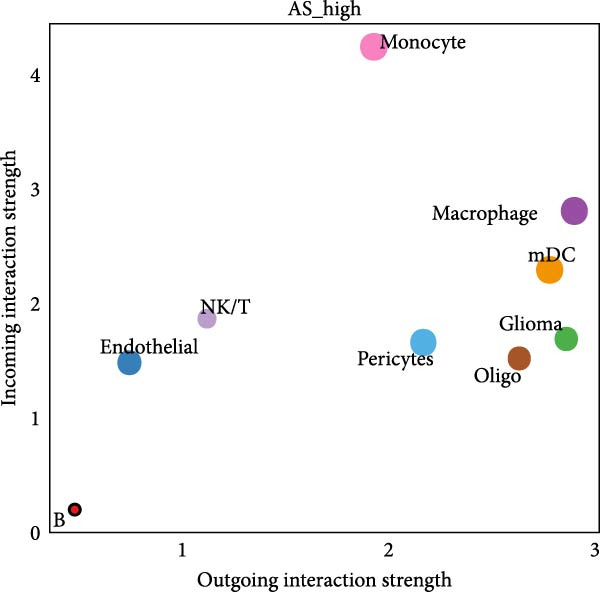
(L)

**Figure:**
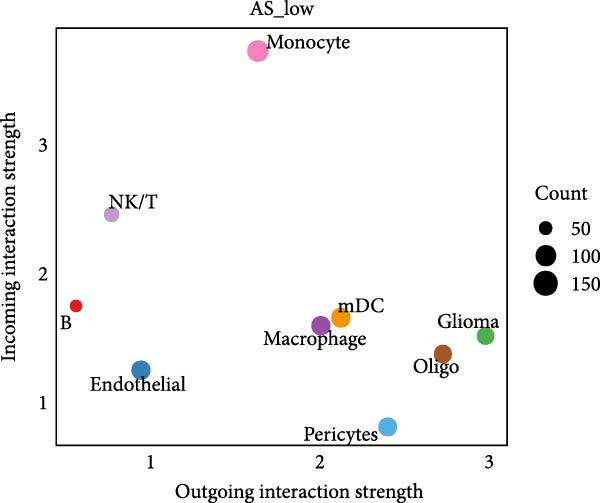
(M)

Similarly, we analyzed the GSE139448 dataset, which contained a total of 13,240 cells, including 12,412 glioma cells, 592 macrophages, 102 MAST cells, 118 endothelial cells, and 16 pericytes (Figure 5D). The risk score results are shown in Figure 5E. Glioma cells and macrophages accounted for the majority of cell types (Figure 5F). Figure 5G–H depicts the AS risk scores for various cell types within the GSE182109 and GSE139448 datasets. Figure 5I–M illustrates the variations in cellular communication between the AS high‐ and low‐risk groups. The heatmap displaying the communication disparities between the high and low AS groups in the GSE182109 single‐cell dataset is presented in Supporting Information 4: Figure S4.

### 3.6. Correlation Analysis of Potential Targets of the AS Model

We performed abundance analysis of potential target proteins of the AS model. As shown in Figure 6A, the proteins were sorted from high to low correlation coefficients: FOSL1, ITGAV, CD44, DLG5, CTNND1, FSCN1, and ELMO2. The abundance correlation analysis results for each protein are shown in Figure 6B–H. Similarly, as shown in Figure 6I–P, we evaluated the association between the AS model and CERES. We evaluated the correlation between the AS model and drugs from the GDSCv2 and CTRP databases to identify potential targets. In the GDSCv2 database, we identified potential targets such as BRD‐K34099515, BRD‐09344309, EX‐527, BRD‐A02303741, isonicotinohydroxamic acid, and myricetin (Figure 6Q). Notable disparities were evident in the anticipated efficacy of these therapeutic targets between the AS high‐ and low‐risk cohorts, with the AS low‐risk cohort demonstrating greater predicted drug effectiveness (Figure 6R). Similarly, in the CTRP database, we identified three drugs: PD03259011060, AZD64822169, and SCH7729841564 (Figure 6S). The predicted effectiveness of these drugs was markedly diminished in the AS high‐risk cohort relative to the AS low‐risk cohort (Figure 6T).

Figure 6Correlation analysis of potential targets of the AS model. (A–H) Correlation between AS and protein abundance. (I–P) Correlation between AS and CERES. (Q–T) Correlation between AS and drugs in GDSCv2 and CTRP databases.

**Figure:**
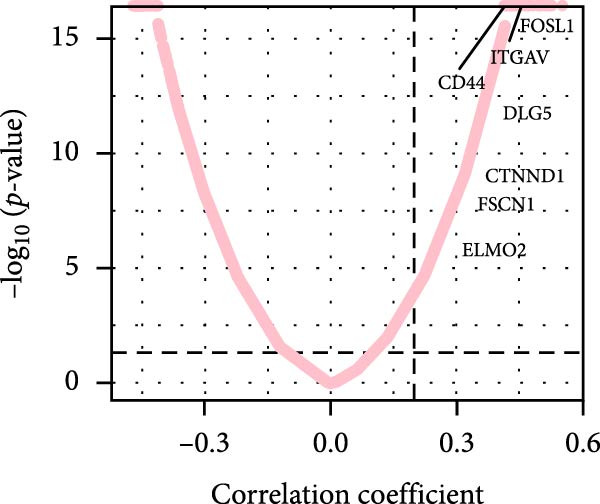
(A)

**Figure:**
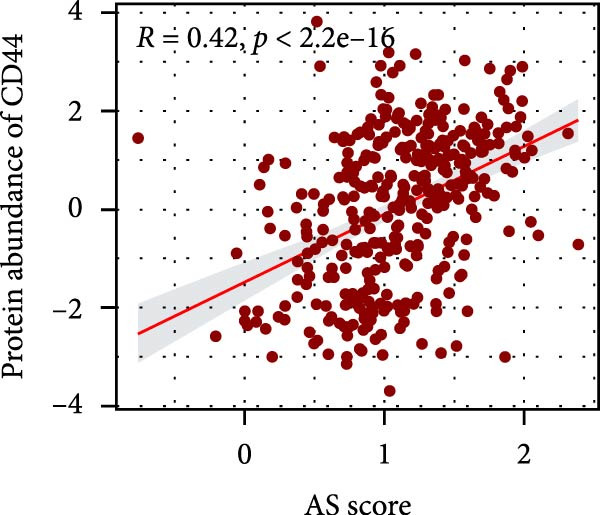
(B)

**Figure:**
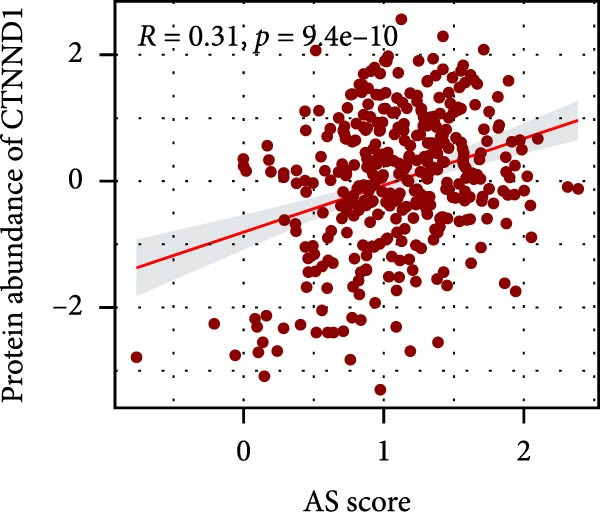
(C)

**Figure:**
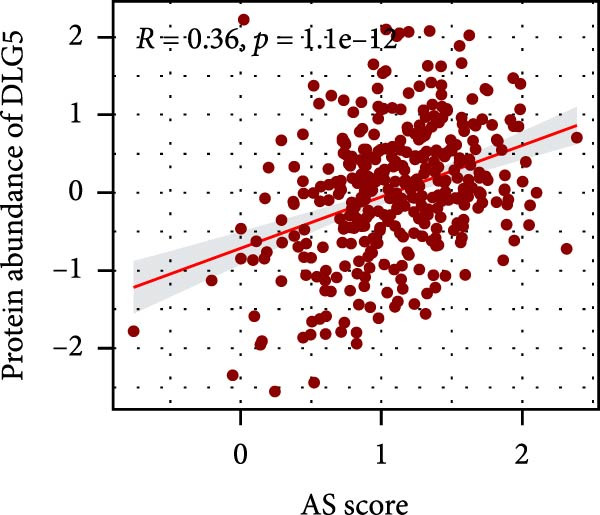
(D)

**Figure:**
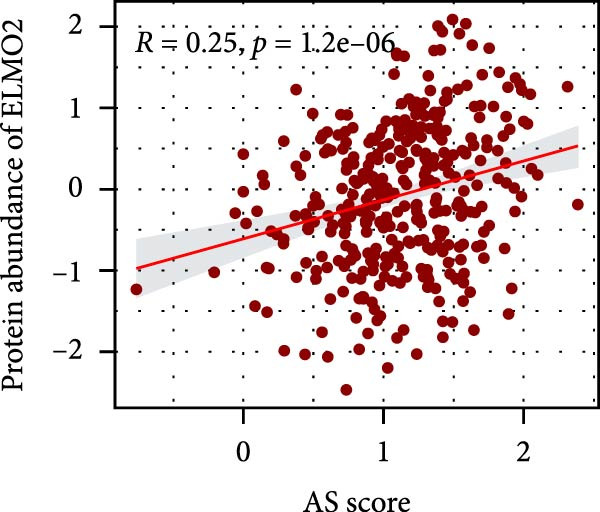
(E)

**Figure:**
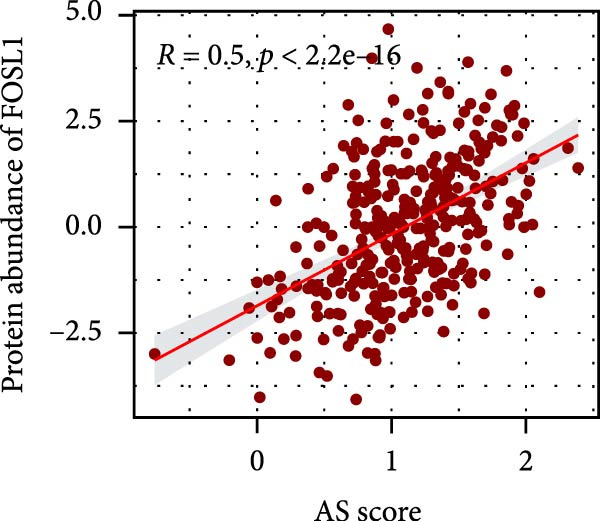
(F)

**Figure:**
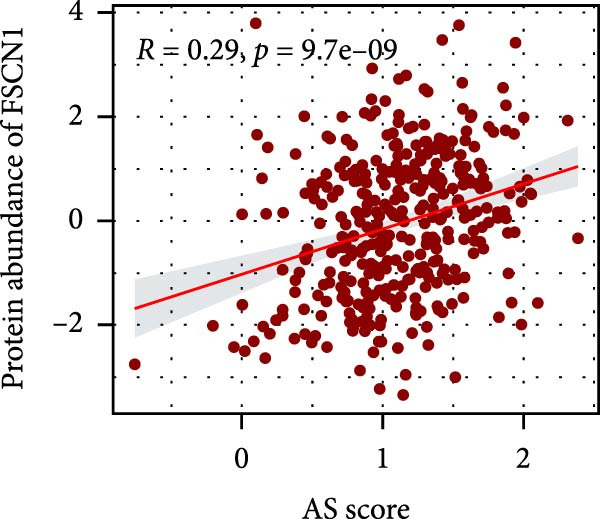
(G)

**Figure:**
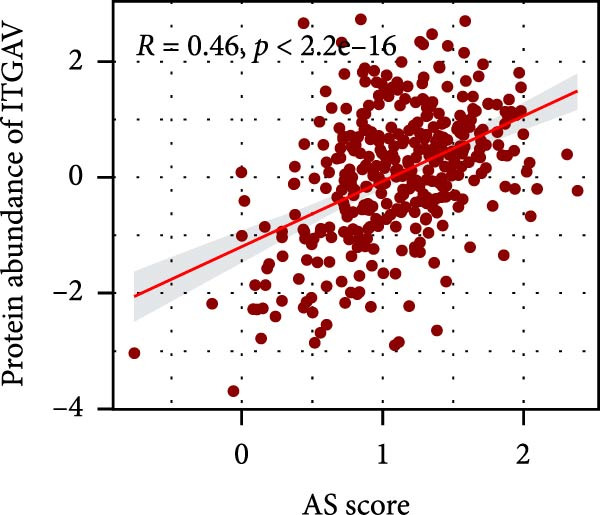
(H)

**Figure:**
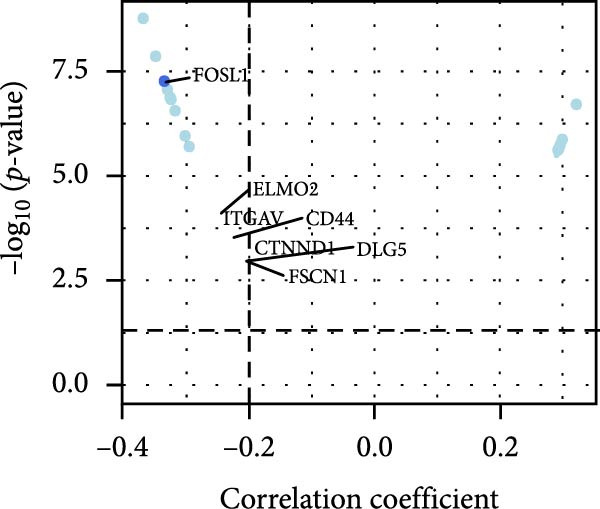
(I)

**Figure:**
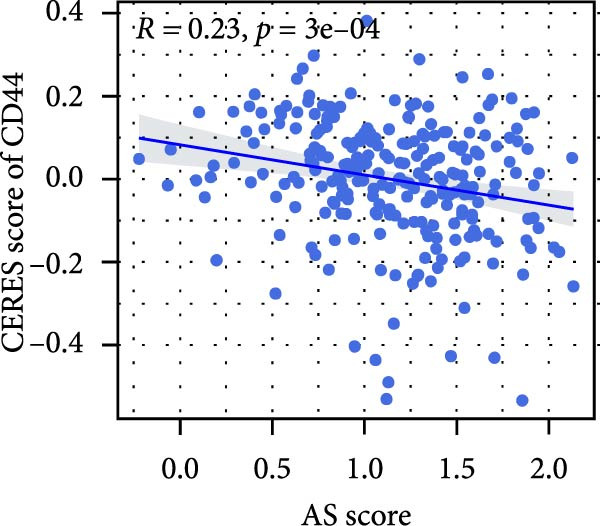
(J)

**Figure:**
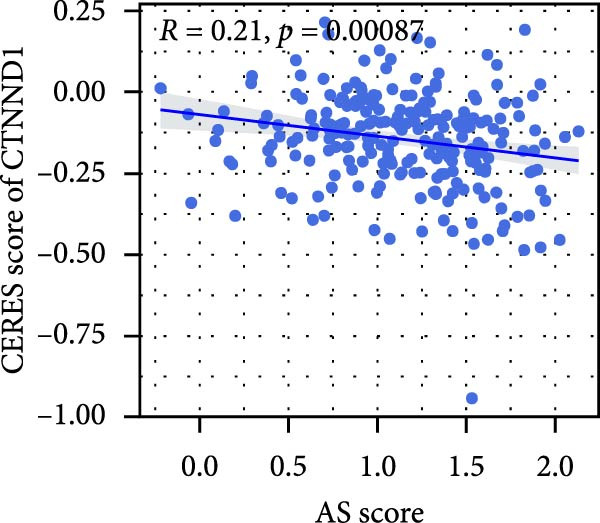
(K)

**Figure:**
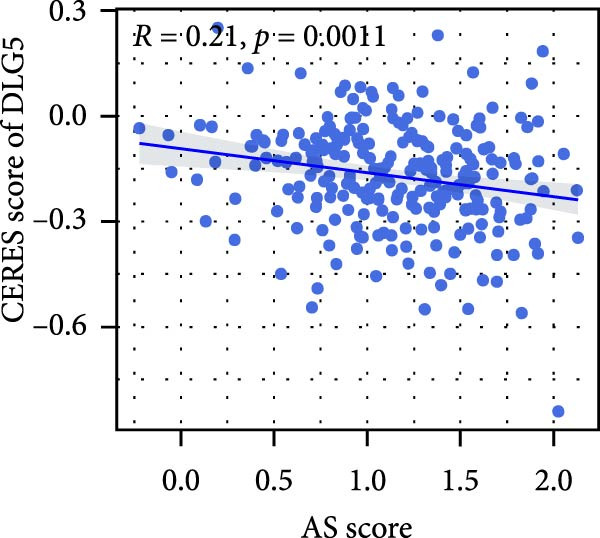
(L)

**Figure:**
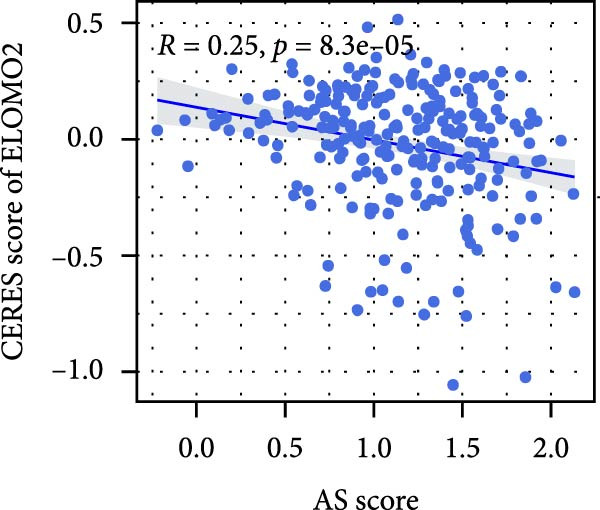
(M)

**Figure:**
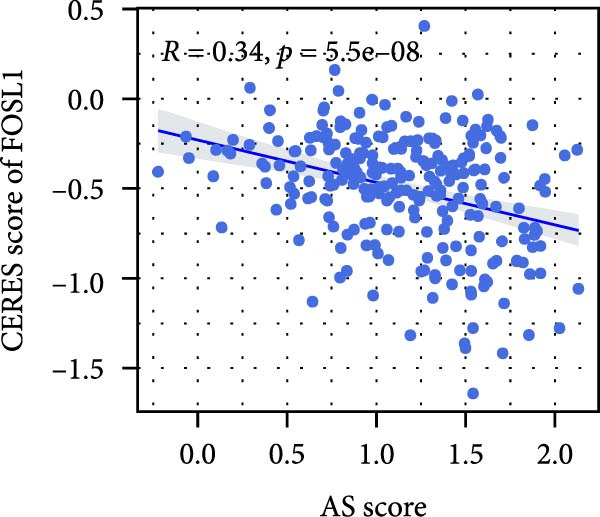
(N)

**Figure:**
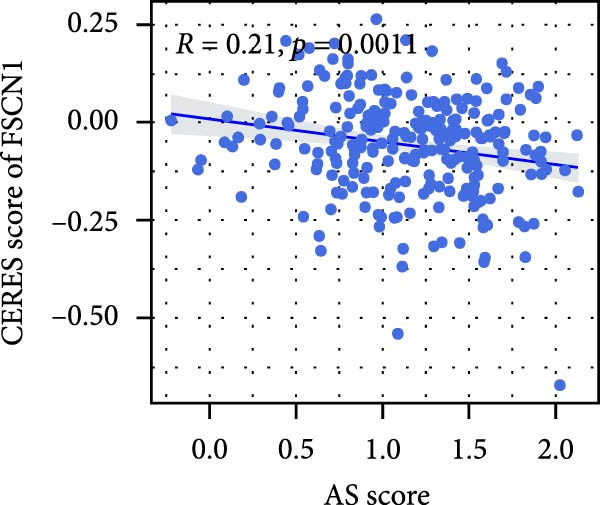
(O)

**Figure:**
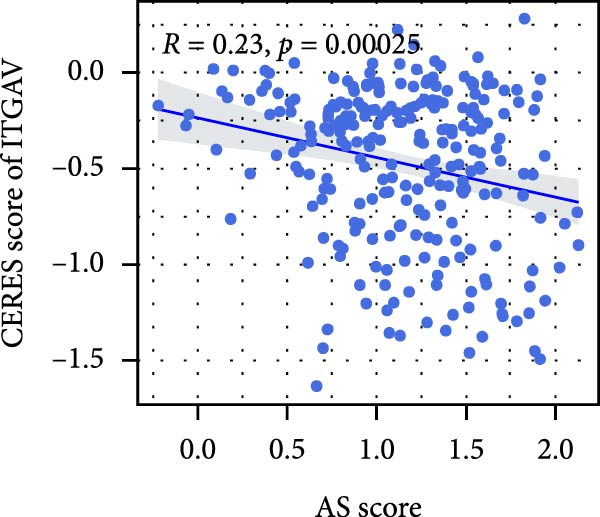
(P)

**Figure:**
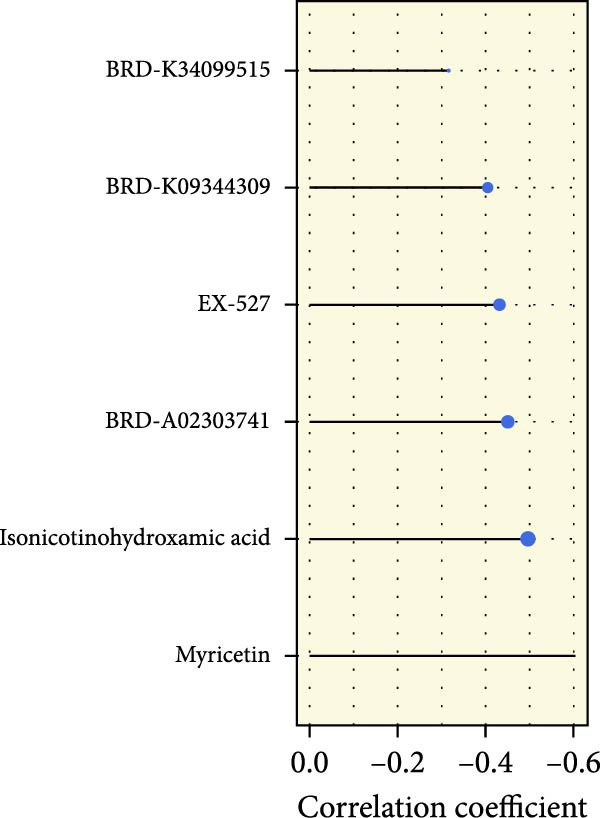
(Q)

**Figure:**
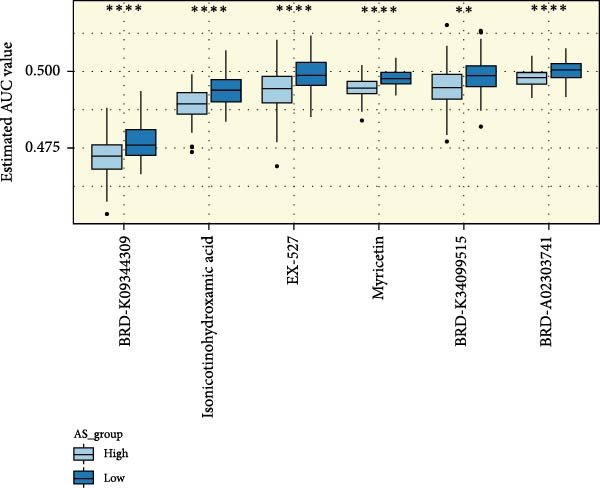
(R)

**Figure:**
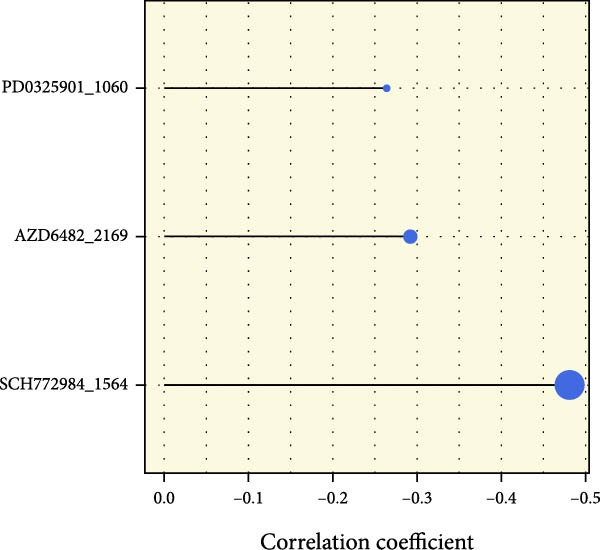
(S)

**Figure:**
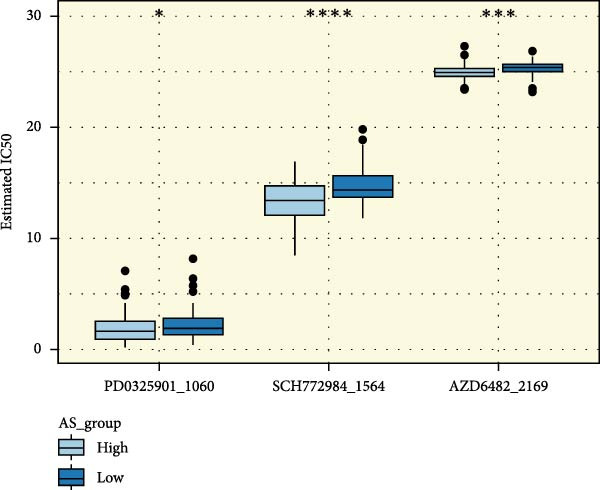
(T)

### 3.7. Analysis of Model Gene HSPB1

We conducted an association study between the gene HSPB1 and the AS model and found that HSPB1 exhibited a positive correlation with the AS model risk (Figure 7A), with *R* = 0.75 and *p*  < 2.2e–16. Additionally, the manifestation intensity of HSPB1 had a good predictive effect on GBM prognosis. As depicted in Figure 7B, individuals with elevated HSPB1 manifestation intensities exhibited markedly reduced survival probabilities compared to those with diminished HSPB1 manifestation intensities. HSPB1 also exhibited differential expression in the immunohistochemistry of the HPA database (Figure 7C, D). Ultimately, we examined the association between HSPB1 manifestation intensities and factors related to tumor immunity, such as immuno‐stimulator, immuno‐inhibitor, MHC, receptor, and chemokine. As shown in Figure 7E, with increasing HSPB1 levels, immune‐related gene pathways exhibited varying degrees of enrichment, which diverged notably from the group with diminished HSPB1 manifestation.

Figure 7Analysis results of model gene HSPB1. (A) Correlation chart between AS and HSPB1. (B) Survival analysis result chart of HSPB1 expression levels. (C, D) Immunohistochemical results of HSPB1 (from HPA database). (E) Heatmap of correlation between HSPB1 and immune‐related genes.

**Figure:**
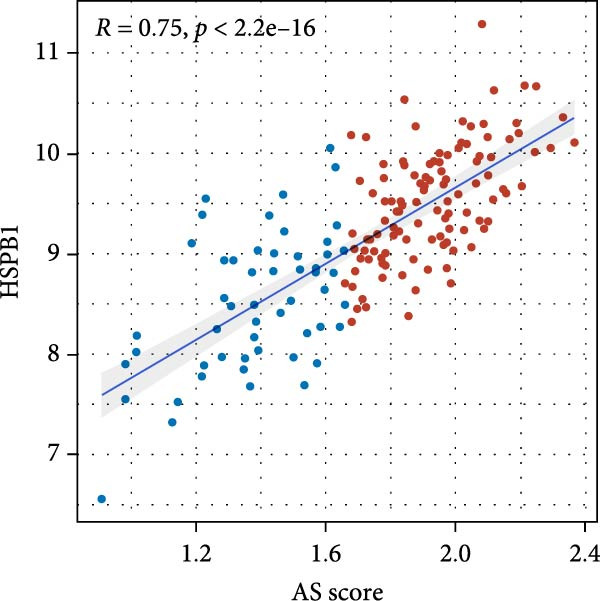
(A)

**Figure:**
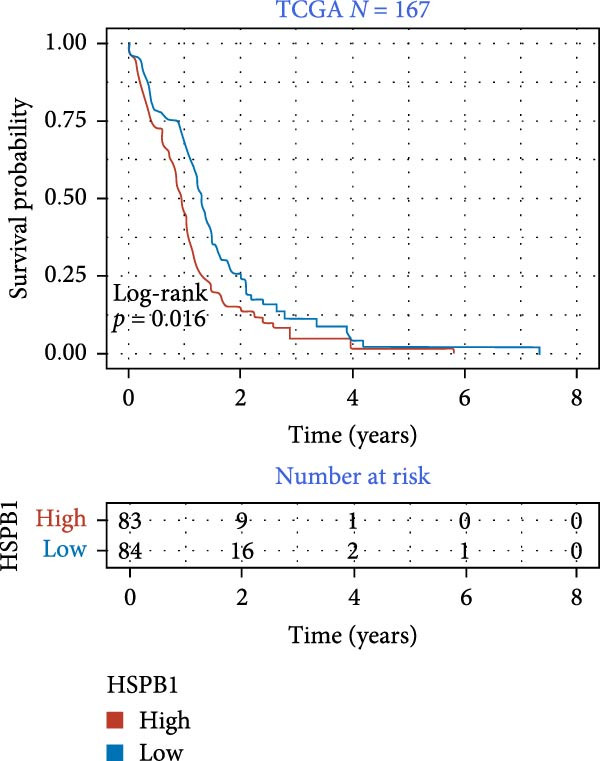
(B)

**Figure:**
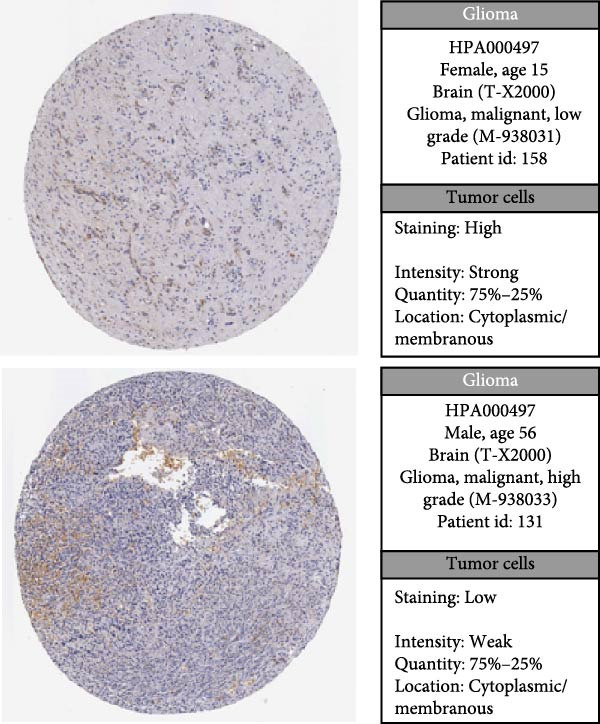
(C)

**Figure:**
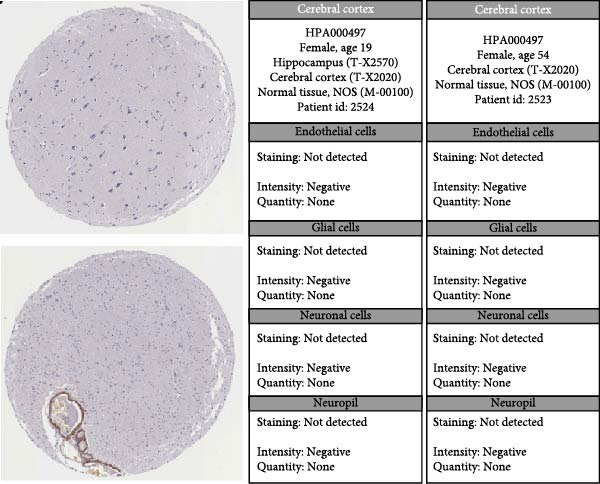
(D)

**Figure:**
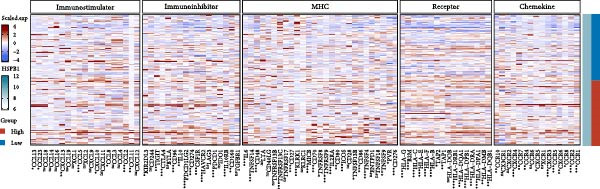
(E)

## 4. Discussion

This research comprehensively examined the expression profiles of genes associated with programed cell death in GBM and their correlation with prognostic outcomes by amalgamating transcriptomic information and single‐cell sequencing data from various repositories, including TCGA, CGGA, and GEO. The results demonstrate that apoptosis‐related genes exhibit significant differential expression between GBM neoplastic tissues and their surrounding non‐neoplastic counterparts, and the manifestation intensities of certain genes are intimately linked to the prognostic trajectory of GBM patients.

Genes such as BRCA1, CHEK2, and IKBKE are significantly upregulated in tumor tissues and serve as prognostic risk factors for GBM patients, suggesting that these genes may promote the initiation and progression of GBM by promoting neoplastic cell multiplication and impeding programed cell death [27]. In GBM, higher BRCA1 positivity is related to shorter survival of patients. Through its role as a transcriptional coactivator of RRM2, high expression of BRCA1 may mask its functional defects, leading to inefficient DNA damage repair, thereby promoting genetic mutations and instability in tumor cells and accelerating tumor progression [28]. Additionally, BRCA1 regulates the G2/M phase checkpoint of the cell cycle, and its overexpression may disrupt cell cycle control, enabling tumor cells to bypass cell cycle arrest and continue to proliferate [29]. CHEK2 in GBM continuously activates the cell cycle checkpoint, allowing tumor cells to enter the cell cycle without DNA damage repair, increasing genomic instability. Furthermore, CHEK2 inhibits cell apoptosis by regulating downstream target genes, such as p53, enabling damaged tumor cells to survive and proliferate [30]. The expression of CHEK2 in human GBMs shows an inverse association with the activation of T‐cells and antigen presentation. The suppression of antitumor immunity by CHEK2 might result from the inactivation of the STING pathway, instead of affecting the DNA‐damage response [31]. Moreover, elevated levels of IKBKE perpetuate the stimulation of the NF‐κB signal transduction cascade, leading to the upregulation of Bcl‐2, which in turn suppresses programed cell death and fosters the survival and multiplication of tumor cells, while also augmenting the migratory and invasive potential of GBM cells [32, 33]. It was reported that IKBKE could exert its oncogenic functions via the IKBKE/HMGA1a/ZEB2 signaling axis in GBM [34].

Conversely, genes such as ZMYND11, MAPK8, and RPS3 exhibit notable upregulation in surrounding non‐neoplastic tissues and function as prognostic safeguarding elements for GBM patients, possibly exerting antineoplastic effects by promoting programed cell death and curbing neoplastic cell multiplication. Research suggests that diminished expression of ZMYND11 is positively associated with the median duration of life in GBM patients, indicating that it may extend patient longevity by impeding neoplastic cell multiplication and infiltration [35, 36]. The decrease of ZMYND11 expression induced by miR‐196a‐5p greatly promoted the proliferation and invasion of GBM cells, causing cell cycle arrest and apoptosis [37]. In adjacent nontumor tissues, high expression of MAPK8 helps maintain normal cell function and stability, preventing stress‐induced cell damage and death, thereby providing a better anti‐TME for the body. MAPK8 is implicated in the infiltration and functional modulation of immune cells, and its elevated expression may facilitate the infiltration of immune cells such as T cells and natural killer cells in surrounding non‐neoplastic tissues, thereby augmenting the body’s immune surveillance and offensive capabilities against neoplasms and impeding tumor expansion and dissemination [38, 39]. Research indicates that rpS3 translocates to the nucleus and undergoes ubiquitination by RING finger protein 138 (RNF138) in GBM cells following radiation exposure, resulting in the degradation of rpS3 and enhancing the radioresistance of GBM cells. Additionally, rpS3 interacts with DNA damage‐inducible transcript 3 (DDIT3), promoting DDIT3‐mediated apoptosis, especially in ΔRNF138 GBM cells. This suggests that the interaction between RNF138 and rpS3 may represent a potential therapeutic target for GBM [40].

Further GO and KEGG enrichment examinations disclosed that these apoptosis‐related genes, closely associated with prognosis, are primarily enriched in apoptosis signaling pathways and virus infection‐related pathways, suggesting that apoptosis‐related genes may affect the biological behavior of GBM by participating in the regulation of these signaling pathways [41–43]. Additionally, this study found that apoptosis‐related genes closely related to prognosis exhibit amplifications or deletions on different chromosomes, which may be associated with abnormal gene expression, providing clues for subsequent molecular mechanism research.

In terms of constructing the prognostic model, this study employed 101 machine learning algorithms [44] and ultimately determined that the CoxBoost + ridge model is the best model. This model exhibits commendable predictive efficacy across various datasets and has a higher *C*‐index value compared to traditional prognostic factors, such as age and gender, suggesting its high clinical application value. Furthermore, by comparing with models reported in other literature, the apoptosis‐related gene prognostic model (AS model) constructed in this study is not inferior to most prediction models in terms of *C*‐index value, further verifying its superior predictive performance.

In the examination of tumor immunity, this study identified notable disparities in tumor immune infiltration and the activation of pathways related to immunity between the high‐ and low‐risk groups in the AS model. For instance, there are marked differences in the infiltration of immune cells such as plasma cells, macrophage TAMs, and MDSCs between the high‐ and low‐risk AS groups, suggesting that the AS model may influence the prognostic outcome of GBM patients by modulating the tumor immune microenvironment. Furthermore, there are also significant variations in the activation of immune‐related pathways between the high‐ and low‐risk AS groups. The predicted immune response using the TIDE algorithm also indicates that there are substantial differences in immunotherapy responses between the high‐ and low‐risk AS groups, suggesting that the AS model may serve as a potential metric for evaluating immunotherapy responses in GBM patients.

In terms of AS analysis of single‐cell data, this study analyzed two single‐cell sequencing datasets, GSE182109 and GSE139448, and found that the risk scores of the AS model differ across different cell types, with glioma cells and macrophages playing crucial roles in the AS model. Furthermore, the differences in cell communication between high‐ and low‐risk AS groups calculated by CellChat software suggest that the AS model may influence the biological behavior of GBM by affecting intercellular communication. In the analysis of potential targets, this study conducted abundance analysis on potential target proteins of the AS model and evaluated the correlation between the AS model and drugs in the CERES, GDSCv2, and CTRP databases, screening out potential drug targets. The identification of these prospective targets offers fresh avenues for subsequent pharmaceutical development and refinement of therapeutic approaches [45].

Ultimately, we observed a positive correlation between HSPB1 and the risk associated with the AS model, suggesting that heightened HSPB1 expression is intimately linked to an unfavorable prognosis in GBM patients, aligning with recent research conclusions [46, 47]. HSPB1 impedes apoptosis in glioma cells by suppressing the activation of caspase‐3 [48]. Caspase‐3 serves as a pivotal mediator in the process of programed cell death, and its enhanced activity facilitates apoptosis [49]. Studies have shown that drugs such as rosmarinic acid and quercetin can effectively silence HSPB1 expression and induce glioma cell apoptosis by activating the caspase‐3 pathway [50]. HSPB1 maintains NADPH and pentose production in neuroglioma cells by enhancing SIRT2‐mediated G6PD activation, thereby safeguarding cells against oxidative and DNA damage‐related stressors. This antioxidant effect helps glioma cells remain stable in the face of oxidative stress and reduces cell apoptosis caused by oxidative damage [51].

However, research also has limitations. First, the samples are mainly from public databases, and multicenter clinical data should be used for the validation of the model of this study, which fully reflects the complexity of the TME. Second, although the model has excellent prediction performance, it has not been verified in clinical practice, and its application feasibility still needs to be further explored. Future analysis should include the comparison of clinical characteristics of patients with different mutation groups in survival analysis to explore the applicability difference of models in specific subtypes.

## 5. Conclusion

By amalgamating multiomics datasets, this investigation thoroughly analyzed the manifestation patterns of genes implicated in programed cell death in GBM and their association with prognostic outcomes, creating an AS model with notable predictive accuracy, and uncovering the intimate connections between the AS model and the tumor’s immunological milieu, cellular interactions, and so forth. These discoveries not only shed novel light on the etiology of GBM but also present innovative concepts and prospective targets for the clinical assessment, management, and prognostic evaluation of GBM.

## Conflicts of Interest

The authors declare no conflicts of interest.

## Author Contributions

Hailong Wang designed the study and wrote the manuscript, Yansong Lu reviewed the manuscript.

## Funding

This article was not funded.

## Supporting Information

Additional supporting information can be found online in the Supporting Information section.

## Supporting information


**Supporting Information 1** Figure S1. (A) PCA plots of TCGA and GTEx data before and after batch effect removal. (B) Heatmap of expression levels of modeling genes.

**Supporting Information 2** Figure S2. (A–G) Functional analysis of AS with Msigdb Gene Sets. (H) Correlation analysis between AS and TIP tumor immunity and immunotherapy pathways.

**Supporting Information 3** Figure S3. (A‐B) CNV results of TCGA CNV segment data visualized separately for high AS and low AS groups using gistic2.0 software. (C) Combined visualization of mutation results calculated by maftools software based on TCGA mutation data (mutation part) and CNV results from gistic2.0 software (CNV part) using ComplexHeatmap. (D) Differences in CNV Gain or Loss between high and low AS groups from Broad and Focal perspectives using CNV results from gistic2.0 software. (E) Survival analysis results of four prognostic groups obtained by combining TMB values calculated by maftools software based on TCGA mutation data with high and low AS groups.

**Supporting Information 4** Figure S4. Heatmap illustrating the divergence in communication between the high AS and low AS cohorts within the GSE182109 single‐cell dataset.

## Data Availability

The data that support the findings of this study are available from the corresponding author upon reasonable request.
